# Transitional changes in the CRP structure lead to the exposure of proinflammatory binding sites

**DOI:** 10.1038/ncomms14188

**Published:** 2017-01-23

**Authors:** David Braig, Tracy L. Nero, Hans-Georg Koch, Benedict Kaiser, Xiaowei Wang, Jan R. Thiele, Craig J. Morton, Johannes Zeller, Jurij Kiefer, Lawrence A. Potempa, Natalie A. Mellett, Luke A. Miles, Xiao-Jun Du, Peter J. Meikle, Markus Huber-Lang, G. Björn Stark, Michael W. Parker, Karlheinz Peter, Steffen U. Eisenhardt

**Affiliations:** 1 Department of Plastic and Hand Surgery, University of Freiburg Medical Centre, Medical Faculty of the University of Freiburg, 79106 Freiburg, Germany; 2 Baker IDI Heart and Diabetes Institute, Melbourne, Victoria 3004, Australia; 3 ACRF Rational Drug Discovery Centre, St Vincent's Institute of Medical Research, Melbourne, Victoria 3065, Australia; 4 Institute for Biochemistry and Molecular Biology and Spemann-Graduate School for Biology and Medicine University of Freiburg, Medical Faculty of the University of Freiburg, 79104 Freiburg, Germany; 5 Departments of Medicine and Immunology, Monash University, Melbourne, Victoria 3800, Australia; 6 College of Pharmacy, Roosevelt University, Schaumburg, Illinois 60173, USA; 7 Department of Biochemistry and Molecular Biology, Bio21 Molecular Science and Biotechnology Institute, University of Melbourne, Melbourne, Victoria 3052, Australia; 8 Department of Traumatology, Hand, Plastic, and Reconstructive Surgery, Center of Surgery, University of Ulm, 89081 Ulm, Germany

## Abstract

C-reactive protein (CRP) concentrations rise in response to tissue injury or infection. Circulating pentameric CRP (pCRP) localizes to damaged tissue where it leads to complement activation and further tissue damage. In-depth knowledge of the pCRP activation mechanism is essential to develop therapeutic strategies to minimize tissue injury. Here we demonstrate that pCRP by binding to cell-derived microvesicles undergoes a structural change without disrupting the pentameric symmetry (pCRP*). pCRP* constitutes the major CRP species in human-inflamed tissue and allows binding of complement factor 1q (C1q) and activation of the classical complement pathway. pCRP*–microvesicle complexes lead to enhanced recruitment of leukocytes to inflamed tissue. A small-molecule inhibitor of pCRP (1,6-bis(phosphocholine)-hexane), which blocks the pCRP–microvesicle interactions, abrogates these proinflammatory effects. Reducing inflammation-mediated tissue injury by therapeutic inhibition might improve the outcome of myocardial infarction, stroke and other inflammatory conditions.

## Abstract (web summary)

C-reactive protein is a pentameric protein secreted by the liver in response to injury and infection. Here Braig *et al*. show that conformational changes in CRP on the surface of monocyte-derived microvesicles enable binding of complement C1q and lead to activation of the complement cascade and aggravation of inflammation.

C-reactive protein (CRP) is a homopentameric protein (pCRP), which is synthesized by the liver in response to tissue injury and inflammation^1^. Although circulating pCRP is not proinflammatory in healthy subjects, it exacerbates existing tissue injury in a complement-dependent manner. This has been shown in animal models of rat myocardial infarction, lipopolysaccharide (LPS)-mediated tissue inflammation and ischemic cerebral injury^2^,^3^,^4^,^5^,^6^.

The disc-shaped pCRP consists of five identical subunits: each subunit weighs ∼23 kDa. The subunits are non-covalently bound by numerous electrostatic and hydrophobic interactions^7^. The two exposed faces of the pentamer are called the A face (or the ‘effector' face) and the B face (or the ‘binding' face), respectively. The B face binds damaged or apoptotic cell membranes and bacterial cell walls. Residues on the A face of pCRP are known to interact with complement factor 1q (C1q) and the Fcγ receptors; however, the location of these residues in the circulating pentamer suggests that this is not the interacting form of CRP^7^,^8^,^9^,^10^,^11^,^12^.

pCRP localizes to injured tissue where it undergoes conformational changes (that is, formation of monomeric subunits, monomeric human CRP (mCRP)) leading to neoepitope exposure (that is, residues 199–206 become accessible to conformation-specific antibodies 9C9 or 3H12) and complement activation. An initial structural change in the pentameric protein produces pCRP*, a CRP isoform expressing the neoepitope while maintaining an overall pentameric configuration. pCRP* can then dissociate into neoepitope-expressing mCRP^4^,^11^,^13^. Deposits of neoepitope-expressing CRP have been found in various conditions of inflammation. Conformation-specific antibodies are commonly used to detect neoepitope-expressing CRP; however, they cannot distinguish between pCRP* and mCRP^14^,^15^,^16^,^17^,^18^,^19^.

Although the link of CRP tissue deposits, complement activation and increased leukocyte infiltration is well established, little is known about the sequence of molecular interactions that take place in injured tissue. We therefore investigated the interplay of circulating pCRP with human monocytes and closely followed structural changes and protein interactions to identify potential targets for the reduction of CRP-mediated tissue injury. We demonstrate that pCRP binds to LPS-activated monocytes, and is subsequently released on microvesicles where it undergoes structural changes while maintaining pentameric symmetry (pCRP*). Our data further show that pCRP* constitutes the major CRP species in human-inflamed tissue and activates the complement system, which exacerbates the inflammatory response. 1,6-bis(phosphocholine)-hexane (1,6-bis-PC), a small-molecule inhibitor that binds to the pCRP phosphocholine binding site^20^, can inhibit the pCRP–microvesicle interactions and thereby abrogate CRP-mediated tissue injury.

## Results

### pCRP binds to activated monocytes

**Figure 1:**
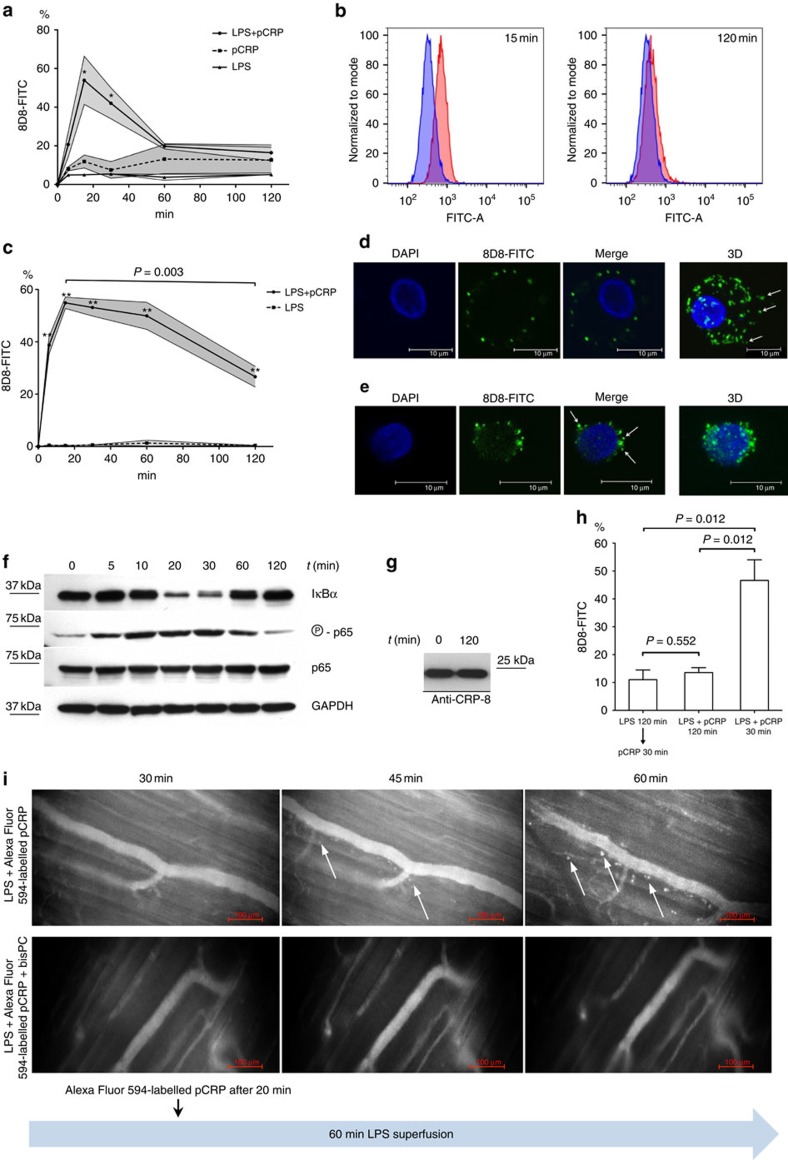
pCRP binds to activated monocytes. (**a**) THP-1 cells were incubated with LPS. Where indicated pCRP (100 μg ml^−1^) was added simultaneously with LPS. Cells were stained with anti-pCRP-8D8/FITC. Fluorescence was recorded by flow cytometry. *P value<0.05 compared to resting cells (paired *t*-test). Shaded areas display the s.e.m. (*n*≥3 for each group and time point). (**b**) Representative fluorograms of LPS-stimulated THP-1 cells in the presence of pCRP at *t*=0 (blue curves) and at indicated time points (red curves). (**c**) pCRP binding to human monocytes was analysed as in (**a**). **P value <0.01 (paired *t*-test). Displayed are means±s.e.m. (*n*=3 for each group and time point). Shaded areas define the s.e.m. (**d**) Confocal fluorescence microscopy of LPS-activated human monocytes in the presence of pCRP. Depicted is a plane from the centre of the cell and a 3D reconstruction of the whole cell. The nucleus was stained with DAPI (blue) and pCRP with anti-pCRP-8D8/FITC (green). pCRP localizes in clusters on the plasma membrane. pCRP–microvesicle complexes separate from the membrane (arrows). Scale bars, 10 μm. (**e**) Confocal fluorescence microscopy of LPS-activated THP-1 cells. Cells were treated as in (**d**). Scale bars, 10 μm. (**f**) Western blot of NF-κB pathway screen of LPS-activated THP-1 cells. Cells were incubated for indicated time periods with LPS, lysed and separated on SDS–PAGE. Uncropped images of western blots are shown in Supplementary Fig. 12. (**g**) Western blot of cell-free supernatants after incubation of THP-1 cells with LPS and pCRP for indicated time periods. CRP was detected after separation on SDS–PAGE, which induced dissociation of the pentamer. The detected size thus corresponds to a CRP monomer (23 kDa). (**h**) pCRP binding to THP-1 cells. Cells were treated as described under (**a**); however, one sample was simultaneously incubated with pCRP and LPS for 120 min, whereas the other sample was first incubated with LPS for 120 min and only then with pCRP for 30 min. Bars indicate mean±s.e.m. *P* values were calculated with a paired *t*-test (*n*=3). (**i**) *In vivo* visualization of the pCRP–leukocyte interaction. Rat cremasteric muscle tissue was stimulated via superfusion with 1 μg ml^−1^ LPS. Alexa Fluor 594-labelled pCRP (25 μg ml^−1^ serum concentration) or pCRP preincubated with 1,6-bis-PC (bisPC) was added 20 min later. At *t*=45 min binding of pCRP to transmigrated leukocytes can be observed (arrows). In the presence of 1,6-bis-PC, labelled pCRP can be seen in the microcirculation, but not on transmigrating leukocytes. Scale bars, 100 μm.

THP-1 monocytic cells were incubated with LPS and/or pCRP. Binding was quantified by flow cytometry with conformation-specific antibodies (anti-pCRP-8D8). Unstimulated cells bound little pCRP over time. In contrast, LPS-activated cells showed a sharp increase in pCRP attachment, which peaked at 15 min and then gradually decreased over time, until it reached the level of resting cells at about 120 min (Fig. 1a,b). Human monocytes, isolated from peripheral blood of healthy subjects, showed similar binding characteristics (Fig. 1c). The pCRP–monocyte interaction was Ca^2+^-dependent and occurred within physiological pCRP serum concentrations (Supplementary Fig. 1). Overall, these results suggest a specific interaction of pCRP via its Ca^2+^-dependent binding sites with exposed ligands on LPS-activated monocytes.

### pCRP is released on cell-derived microvesicles

Binding was further characterized by confocal fluorescence microscopy, which revealed clusters of pCRP on the cell plasma membrane of activated monocytes and THP-1 cells. Shedding of pCRP-bearing membrane areas was visible and some clusters could be observed in close vicinity to the cells (Fig. 1d,e, arrows). These latter clusters ranged in diameter between 100 and 500 nm, which were the typical size of plasma membrane-derived microvesicles. We will adhere to the convention outlined by Buzas *et al*.^21^ whereby microvesicles are 100–1,000 nm in diameter, exosomes are smaller microvesicles with diameters of 50–100 nm and the larger apoptotic vesicles have diameters of 100–5,000 nm.

We further investigated the time correlation between pCRP binding and activation of the nuclear factor-κB (NF-κB) pathway, which is switched on in LPS-stimulated immune cells. In LPS-stimulated cells, degradation of IκBα and phosphorylation of p65 increased simultaneously with pCRP binding (Fig. 1f). Likewise, when cells entered a quiescence state after 120 min, pCRP was completely released from the cell plasma membrane. Western blots to detect CRP in the cell-free supernatant before (*t*=0 min) and after LPS stimulation (*t*=120 min) confirmed that pCRP was not consumed by the cells (Fig. 1g). To confirm that binding of pCRP requires membrane changes, which are only present in active cells, THP-1 cells were first stimulated with LPS for 120 min and only then incubated with pCRP. Binding in this group was similar to cells that were simultaneously incubated with LPS and pCRP for 120 min (Fig. 1h).

These results indicate that circulating pCRP binds to the perturbed plasma membrane of activated monocytes and is released into the surrounding tissue bound to cell-derived microvesicles.

### Binding of pCRP to activated leukocytes in the microcirculation

To confirm the *in vivo* relevance of our findings, we performed intravital microscopic tracking of pCRP in LPS-induced cremasteric muscle inflammation in rats. Shortly after intravenous injection, the Alexa Fluor 594-labelled pCRP could be detected in the microcirculation. During the course of the inflammation, pCRP bound to transmigrating leukocytes in postcapillary venules and was transported in the inflamed perivascular tissue. If pCRP was preincubated with the small-molecule inhibitor 1,6-bis-PC that blocks the phosphocholine binding sites of pCRP, no transmigrating leukocytes with bound Alexa Fluor 594-labelled pCRP were detected (Fig. 1i).

### Stimulated cells release LPC-enriched microvesicles

**Figure 2:**
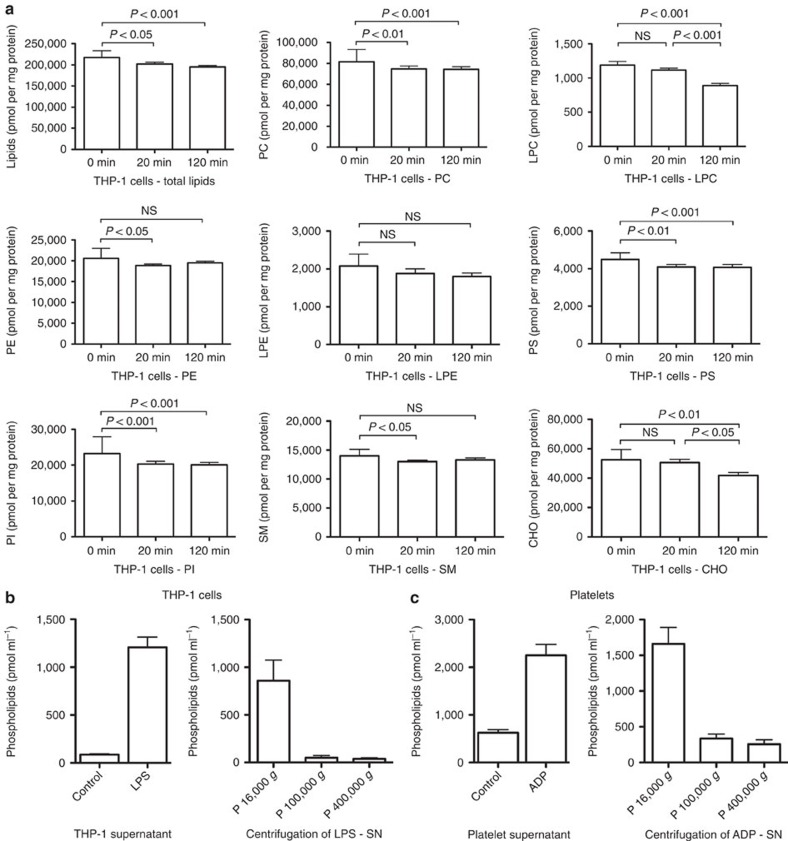
LPS-activated THP-1 cells release microvesicles. THP-1 cells were stimulated with LPS for indicated time periods. Total lipids were extracted and subjected to mass spectrometric analysis. (**a**) THP-1 cells lost significant amounts of lipids after LPS stimulation. Most lipids decreased significantly over time, with LPC and CHO showing the most distinctive decline, which occurred between 20 and 120 min after stimulation. Lipid values are expressed as pmol per mg protein. Other lipids identified were: LPE, phosphatidylcholine (PC), PE, PI, PS and SMs (*n*=12). *P* values were calculated with one-way analysis of variance (ANOVA). (**b**) Lipid concentrations in the 1,500 *g* supernatant of LPS stimulated and resting THP-1 cells were determined by mass spectrometric analysis (*n*=6). The 1,500 *g* supernatant after LPS stimulation was further separated by differential centrifugation. Microvesicles were collected at 16,000 *g*, and smaller microvesicles pelleted at 100,000 *g* and 400,000 *g*. Displayed lipid values are expressed as pmol ml^−1^ supernatant (SN) and the s.d. (*n*=6). (**c**) Lipid concentrations in the 1,500 *g* supernatant of ADP-stimulated and resting platelets (*n*=2). The 1,500 *g* supernatant after ADP stimulation was further separated by differential centrifugation as described in (**b**). Displayed lipid values are expressed in pmol ml^−1^ supernatant±s.d. (*n*=2).

To further assess the LPS-induced membrane changes, we determined the lipid composition of LPS-stimulated monocytic cells and cell-derived microvesicles by lipidomics. THP-1 cells were incubated with LPS for different time periods, total lipids extracted and analysed by tandem mass spectrometry. Most lipids in the cells decreased over time; the decrease was most pronounced for lysophosphatidylcholine (LPC) and cholesterol (CHO), which dropped significantly between 20 and 120 min after stimulation. This corresponds to the decrement of pCRP attachment and microvesicle release (Fig. 2a and Supplementary Fig. 2A). As loss of lipids might be due to shedding of microvesicles, we examined the lipid content of the 1,500 *g* cell-free supernatant of resting and LPS-activated cells. The supernatant fluids of unstimulated cells contained only 87±3 pmol ml^−1^ of phospholipids, whereas 1,207±44 pmol ml^−1^ could be detected in the LPS-stimulated group (Fig. 2b). Subsequent differential centrifugation of the 1,500 *g* supernatant revealed that the majority of microvesicles could be pelleted at 16,000 *g*. A minor species of even smaller vesicles could be pelleted from the 16,000 *g* supernatant at 100,000 *g*, as well as at 400,000 *g* (Fig. 2b). We observed similar results with ADP-stimulated platelets. The supernatant of unstimulated platelets contained 626±5 pmol ml^−1^ of phospholipids, whereas 2,252±18 pmol ml^−1^ were detected after stimulation. Again, differential centrifugation pelleted most lipids at 16,000 *g* and smaller fractions at 100,000 *g* and 400,000 *g* (Fig. 2c).

**Figure 3:**
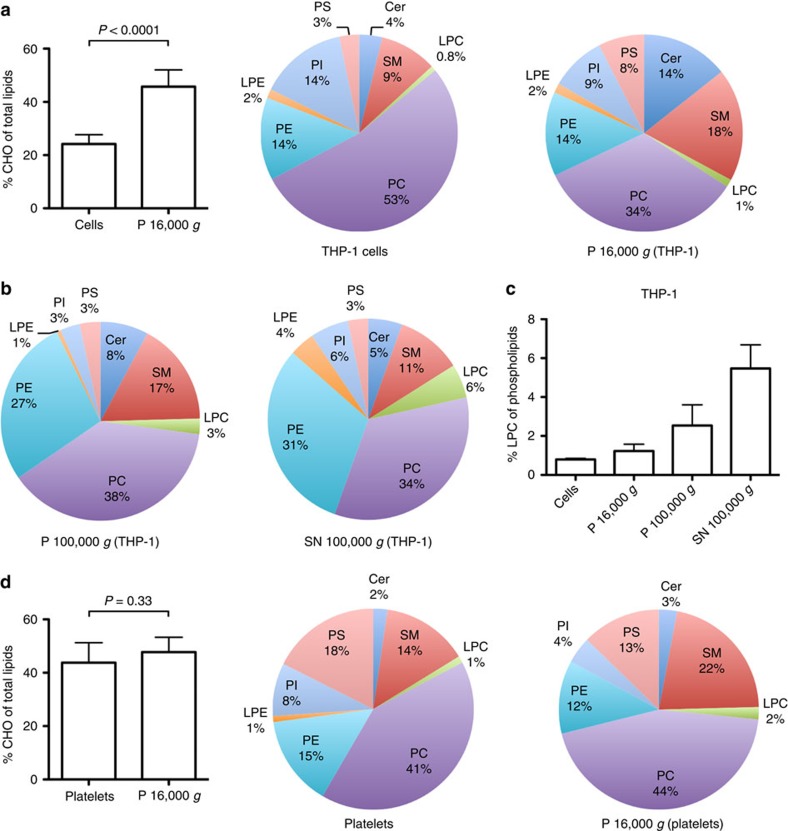
Lipid composition of THP-1 cells and microvesicles. (**a**) THP-1 cells and cell-derived microvesicles (16,000 *g* pellet) were lipid extracted and subjected to mass spectrometric analysis. Microvesicles contain large amounts of CHO, phosphatidylcholine (PC), SMs, ceramides (Cer) and PS, which are predominantly found in the cell plasma membrane. The other lipids identified were: LPC, LPE, PE and PI. Displayed are means and s.d. *P* values were calculated with an unpaired *t*-test (*n*=12). (**b**) Smaller microvesicles contain increasing amounts of PE, reflecting their endoplasmic reticulum origins. (**c**) LPC content increases in small microvesicles and induces a positive membrane curvature due to the flexibility of the single-chain fatty acid (*n*≥4 for each group). In (**a**), CHO is expressed in % of total lipids. Other membrane lipids are expressed as per cent of total lipids (excluding CHO). Lipids, which comprises <0.5% of total lipids, were excluded from the analysis. (**d**) The lipid composition of platelets and platelet-derived microvesicles (16,000 *g*) was determined as described above (**a**–**c**) (*n*=6). *P* values were calculated with an unpaired *t*-test.

Determination of the lipid composition of microvesicle populations revealed considerable differences when compared with THP-1 cells, as well as among the individual microvesicle species. Microvesicles in the 16,000 *g* pellet were highly enriched in CHO and the plasma membrane phospholipids sphingomyelin (SM), ceramides (Cer) and phosphatidylserine (PS), when compared to THP-1 cells (Fig. 3a). Their size and composition are characteristic for microvesicles, which are released from the cell plasma membrane^22^. The 100,000 *g* pellet and 100,000 *g* supernatant contained high amounts of phosphatidylethanolamine (PE) and lower amounts of PS, SM and Cer, when compared to microvesicles in the 16,000 *g* pellet (Fig. 3b). In addition, these smaller microvesicles contained increased amounts of LPC (Fig. 3c). This composition reflects their smaller size and indicates their origin from the endoplasmic reticulum. Platelet-derived microvesicles contained a similar CHO content, were enriched in SM, but contained less phosphatidylinositol (PI) than platelets (Fig. 3d). Again, the smaller vesicles contained increased amounts of LPC (Supplementary Fig. 2B).

We further determined the influence of pCRP on microvesicle release by flow cytometry. Microvesicles were identified by their characteristic size and PS content in the outer membrane leaflet^23^. THP-1 cells rapidly released microvesicles after LPS stimulation and no further increase could be detected after 120 min. There was no significant difference between LPS and LPS+pCRP-treated cells. pCRP alone failed to induce microvesicle release (Supplementary Fig. 2C). Microvesicle production was due to cell activation and not the result of cell apoptosis, as no increase in Annexin V binding to LPS-treated cells was observed (Supplementary Fig. 2D). We additionally differentiated human monocytes into M1 and M2 macrophages and analysed the ability of these macrophages to release microvesicles on stimulation with phorbol 12-myristate 13-acetate (PMA), monophosphoryl lipid A (MPLA) and LPS (Supplementary Fig. 2E,F). In contrast to monocytes and platelets, which show a marked increase of microvesicle release on stimulation, both macrophage subsets showed a constant release of microvesicles over time, which did not increase on stimulation.

### pCRP binding to microvesicles induces structural changes

We analysed the interaction of pCRP with microvesicles in more detail. pCRP was incubated with either THP-1 or Jurkat cell-derived microvesicles. Bound pCRP was pelleted and analysed by blue native–polyacrylamide gel electrophoresis (BN–PAGE).

**Figure 4:**
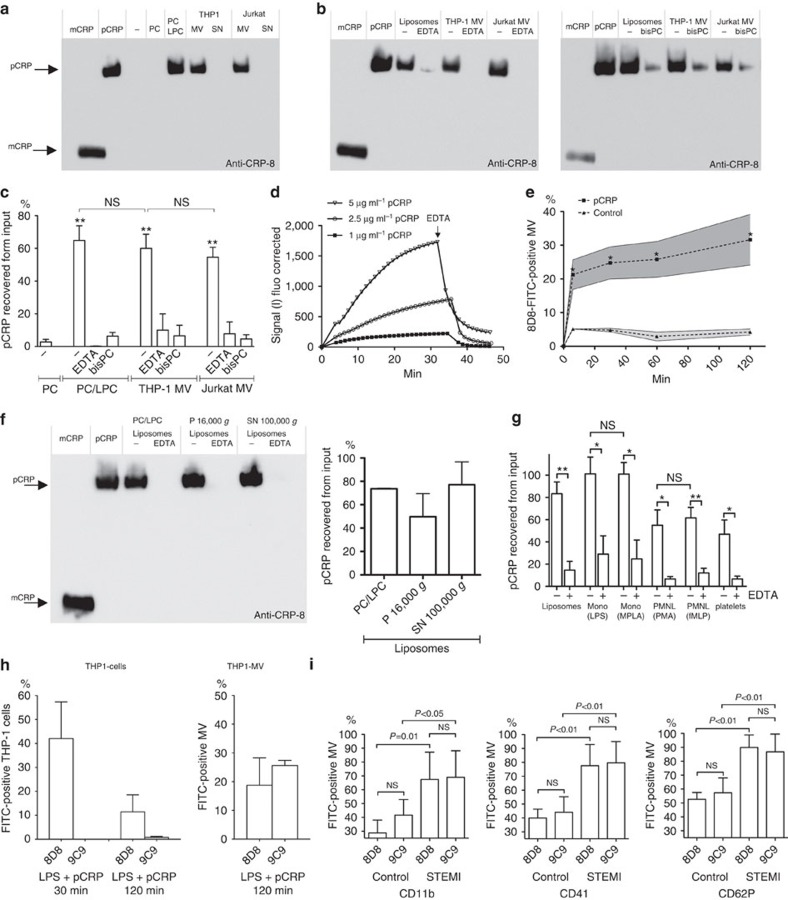
pCRP binding to microvesicles induces structural changes. pCRP was incubated with liposomes (PC or PC/LPC) and microvesicles (MV) from THP-1 and Jurkat cells. The supernatant (SN) of the last wash step after MV purification was used as control. Vesicles were pelleted and subjected to BN–PAGE. (**a**) pCRP bound to PC/LPC liposomes, THP-1 and Jurkat cell-derived MV. No binding occurred to PC-only liposomes and no CRP could be found in the pellet fraction of the supernatant. mCRP was not found on the surface of MV and liposomes. (**b**) Ca^2+^-depletion (EDTA) or 1,6-bis-PC (bisPC) reduces binding of pCRP to MV. (**c**) Quantification of pCRP binding. There was no significant difference in the amount of pCRP that bound to either MV species or PC/LPC liposomes (*n*=5). EDTA and 1,6-bis-PC significantly reduced binding (*n*=2). *P* values were calculated with a Student's *t*-test. **P value of <0.01, NS=not significant. Displayed are means and s.e.m. (**d**) Kinetics of the pCRP–MV interaction were determined by measuring binding of Alexa Fluor 488-labelled pCRP to immobilized THP-1 MV. (**e**) Binding of pCRP to THP-1 cell-derived MV was analysed by flow cytometry with conformation-specific antibodies for pCRP (anti-pCRP-8D8/FITC). *P* values were calculated with a paired *t*-test. *P value of <0.05 to control MV and shaded areas display the s.e.m. (*n*=4). (**f**) Binding of pCRP to liposome mimics of THP-1 MV (P 16,000 *g* and SN 100,000 *g*) was analysed as described above. Binding was Ca^2+^-dependent and we did not observe dissociation of pCRP into its monomeric subunits. Displayed are means and s.e.m. (*n*=3). (**g**) Binding of pCRP to different cell-derived MV was analysed on BN–PAGE as described in (**c**). Monocytes (Mono), polymorphonuclear leukocytes (PMNL), phorbol 12-myristate 13-acetate (PMA), monophosphoryl lipid A (MPLA), *N*-formylmethionyl-leucyl-phenylalanine (fMLP). Displayed are means and s.e.m. (*n*=3). *P* values were calculated with a Student's *t*-test. *P value <0.05 and **P value <0.01. (**h**) Conformational changes in pCRP on binding to THP-1 cells and MV were assessed with conformation-specific antibodies by flow cytometry. CRP bound to THP-1 cells could only be detected by anti-pCRP-8D8 antibodies, whereas CRP on MV was recognized by anti-pCRP-8D8 and anti-mCRP-9C9 antibodies. Displayed are means and s.d. (*n*=3). (**i**) Deposition of CRP on MV of different cellular origin in the circulation of patients with ST-elevation myocardial infarction (STEMI) was analysed by FACS with anti-pCRP-8D8/FITC and anti-mCRP-9C9/FITC antibodies. Displayed are the percentages of FITC-positive MV of each subset. Bars indicate means and s.d. (*n*=4). *P* values were calculated with an unpaired *t*-test.

pCRP can attach to exposed PC and PE head groups of perturbed membrane phospholipids. Introducing LPC into the lipid bilayer of liposomes can mimic this phenomenon^24^. As expected, pCRP bound to LPC containing liposomes but not to PC-only liposomes (Fig. 4a). We also observed pCRP binding to THP-1 and Jurkat cell-derived microvesicles (Fig. 4a). EDTA, as well as 1,6-bis-PC, inhibited the interaction (Fig. 4b), which suggests a Ca^2+^-dependent interaction via the phosphocholine binding sites with membrane phospholipids. BN–PAGE allows separation of pCRP and mCRP. Bound CRP migrated at the same velocity as control pCRP, suggesting that it maintained pentameric symmetry while bound to microvesicles (Fig. 4a,b).

As THP-1 cells harbour Fcγ receptors, which might provide an additional interaction site for CRP on THP-1 cell-derived microvesicles, binding was further quantified on Jurkat cell-derived microvesicles. This T-cell-derived cell line does not harbour Fcγ receptors (Supplementary Fig. 3A,B). There was no significant difference in the amount of pCRP that bound to liposomes, THP-1 or Jurkat cell-derived microvesicles. This suggests the perturbed phospholipid membrane as the primary binding site for pCRP on microvesicles (Fig. 4c).

The dynamics of the pCRP–microvesicle interaction was characterized in real time based on repeated measurements of Alexa Fluor 488-labelled pCRP to immobilized microvesicles. Binding of pCRP to microvesicles was essentially complete after 30 min, and the addition of EDTA led to a rapid dissociation (Fig. 4d).

The release of pCRP–microvesicle complexes by THP-1 cells was examined by flow cytometry. Cells were incubated with LPS and pCRP and the fluorescence of released microvesicles directly quantified with conformation-specific anti-pCRP-8D8 antibodies by gating on the microvesicle population (Fig. 4e and Supplementary Fig. 4A,B). pCRP–microvesicle complexes were detectable within minutes and the fluorescent signal on the vesicles slightly increased over time.

To determine if the lipid composition of microvesicles alone was sufficient to allow binding of pCRP, multilamellar liposomes were created to mimic either large (P 16,000 *g*) or small (SN 100,000 *g*) THP-1 cell-derived microvesicles. These mimics also contained physiological amounts of LPC and lysophosphatidylethanolamine (LPE), as determined by the lipid mass spectrometry experiments. pCRP was found to bind to both microvesicle mimics, and this interaction was EDTA-sensitive (Fig. 4f).

We next tested whether pCRP binds to microvesicles of different cellular original. Microvesicles were purified from stimulated monocytes, polymorphonuclear leukocytes and platelets. All microvesicle species bound pCRP in a Ca^2^-dependent manner, and monocytic microvesicles showed the highest affinity. Binding was independent of the stimulant used to trigger the release of microvesicles (Fig. 4g and Supplementary Fig. 4C,D).

Several reports described conformational changes in pCRP upon membrane binding, which may lead to neoepitope expression or even dissociation of the pentameric pCRP into its subunits^13^,^16^,^25^. Conformational changes on interaction with phospholipid bilayers were monitored by tryptophan fluorescence analysis. Of the six tryptophan residues in each CRP monomer, Trp110 and Trp205 (part of the neoepitope defined by residues 199–206) are buried at the monomer–monomer interface in pCRP. Freshly prepared samples of pCRP, pCRP+liposomes (40% CHO, 40% PC and 20% LPC) and mCRP showed iodide quenching of the tryptophan fluorescence that is in agreement with a simple Stern–Volmer relationship (Supplementary Fig. 5A) which suggests that the tryptophan residues within each protein are all accessible to quenching to the same degree. The lower Stern–Volmer rate constant (*K*_SV_) for mCRP (0.5 M^−1^) indicates that the fluorescence quencher has greater accessibility to the tryptophan residues than in either the pCRP or pCRP+liposome samples (∼0.9 M^−1^), that is, the tryptophan residues are more solvent exposed in mCRP. After incubation overnight to allow full interaction between pCRP and the liposomes^13^, the pCRP and mCRP samples remained in agreement with the simple Stern–Volmer relationship by continuing to show a linear dependence of the *F*_0_/*F* ratio to [I^−^] (Supplementary Fig. 5B). The pCRP+liposome sample, however, has lost the linear relationship and now shows a curved *F*_0_/*F* versus [I^−^] plot. Plotting the data according to the modified Stern–Volmer equation (Supplementary Fig. 5C) indicates that 25% of the tryptophan fluorescence is now accessible to quenching, with the remaining 75% protected. This is consistent with a change in the effective solvent accessibility for at least one tryptophan residue in each monomer within pCRP.

We also analysed neoepitope expression on THP-1 cells and microvesicles with conformation-specific antibodies. As described above (Fig. 1), the anti-pCRP-8D8 antibody detected pCRP on activated monocytic cells. In contrast, anti-mCRP-9C9 antibodies did not detect cell-bound CRP at 30 min and only a minimal signal was observed after 120 min (Fig. 4h). When bound to microvesicles, CRP exhibited reactivity with anti-pCRP-8D8 as well as anti-mCRP-9C9 antibodies (Fig. 4h). In addition, we examined the deposition of native and neoepitope-expressing CRP on the surface of microvesicles from the circulation of patients with ST-elevation myocardial infarction. Microvesicle subsets were identified by CD11b (leukocytes), CD41 (platelets) and CD62P (activated platelets). Similar to the results with purified microvesicles, we identified CRP on microvesicles of different cellular origin, which could be detected by both anti-pCRP-8D8 and anti-mCRP-9C9 antibodies (Fig. 4i). Together with the BN–PAGE data for microvesicle-bound CRP, these data suggest that both pCRP and pCRP* are present on cell-derived microvesicles.

### Structural isoforms of CRP tissue deposits

Tissue-deposited CRP is preferentially recognized by anti-mCRP antibodies (that is, the neoepitope is exposed) and it is therefore thought to be structurally distinct from circulating pCRP (where the neoepitope is not exposed). Most previous studies have not investigated the quaternary structure of deposited CRP and thus the relative proportions of pCRP* and mCRP are unknown^4^,^14^,^15^,^16^,^17^,^26^.

**Figure 5:**
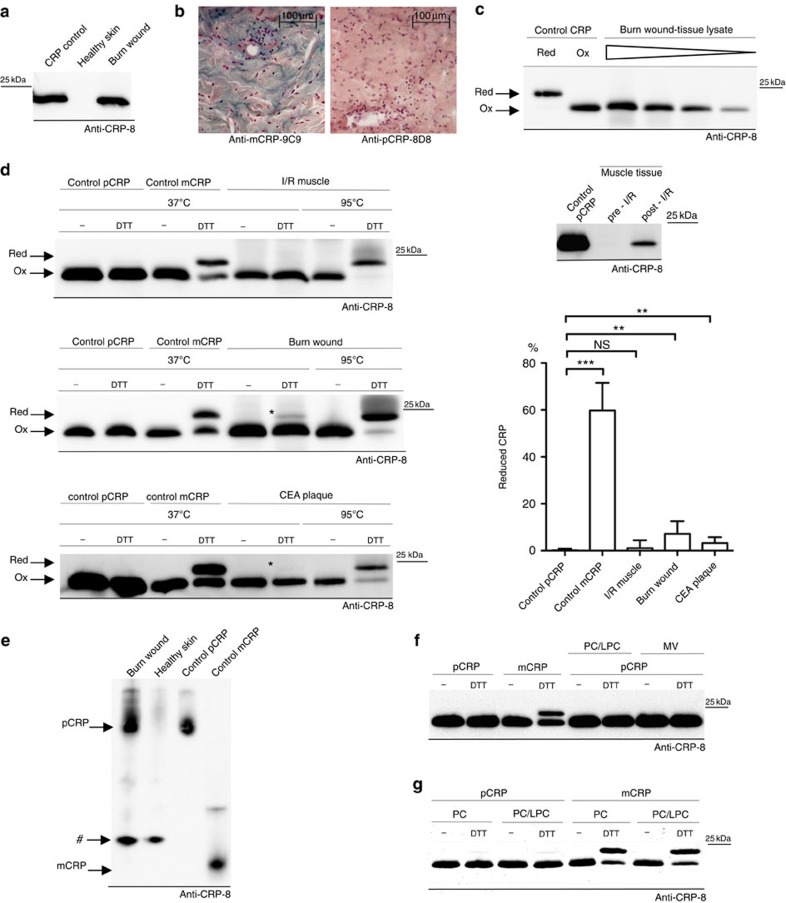
Quaternary structure of CRP in inflamed tissue. (**a**) Tissue lysates of burn wounds and healthy skin were separated on denaturing SDS–PAGE, which induces dissociation of pCRP. Anti-CRP-8 antibodies detected a band in burn wound lysates, which had the size of a CRP monomer (23 kDa). (**b**) Immunohistochemistry of burn wounds revealed distinct staining (green) by anti-mCRP-9C9 antibodies, but only minimal staining with anti-pCRP-8D8 antibodies. Scale bars, 100 μm. (**c**) The oxidation state of CRP in burn wounds was assessed by non-reducing SDS–PAGE. Reduced CRP migrates slower and can thus be distinguished from oxidized CRP. We did not observe reduced CRP in tissue lysates of burn wounds. (**d**) The quaternary structure of CRP in human skeletal muscle after I/R injury (two patients, total of six samples), burn wounds (three patients, total of five samples) and CEA specimens (one patient, total of two samples) was determined by assessing the accessibility of the intrasubunit disulfide bond. Tissue lysates were incubated with 10 mM DTT at 37 °C to assess the accessibility of the intrasubunit disulfide bond. DTT can efficiently reduce the disulfide bond in mCRP, but not in pCRP/pCRP*. Small amounts of tissue-deposited CRP could be reduced by DTT (indicated by an asterisk). Reduction was effective if lysates were heated, which induces pCRP dissociation. Quantification of reduced CRP revealed a significant difference between control pCRP and CRP in tissue lysates of burn wounds and CEA plaques. Displayed are means and s.e.m. *P* values were calculated with an unpaired *t*-test. *P value <0.05, **P value <0.01 and ***P value <0.001. (**e**) Tissue lysates were separated on 1/20 SDS–PAGE. Anti-CRP-8 antibodies recognized a distinct band in burn wound lysates that migrated at the same height as control pCRP. It did not recognize a band with similar size to mCRP. A band, which could be observed in burn wound lysates and healthy skin, was also recognized (#) and most likely reflects binding to a common skin epitope. (**f**) Accessibility of the intrasubunit disulfide bond in CRP in the presence of PC/LPC liposomes and microvesicles (MV). Control mCRP was effectively reduced. No dissociation of pCRP to mCRP was observed. (**g**) Lipid binding does not affect reduction of mCRP by DTT, as reduction of control mCRP incubated with liposomes was unimpeded. Uncropped images of western blots are shown in Supplementary Fig. 13.

As a model of tissue inflammation, we evaluated the previously described CRP deposition in burn wounds^15^ for its quaternary structure and immune reactivity. CRP in burn tissue lysates migrated on denaturing SDS–polyacrylamide gel electrophoresis (SDS–PAGE)-like control CRP at the predicted weight-averaged molecular mass of a CRP monomer (23 kDa) (Fig. 5a). Immunohistochemistry of burn wounds with conformation-specific anti-pCRP-8D8 and anti-mCRP-9C9 antibodies showed distinct staining with anti-mCRP-9C9, but only minimal staining with anti-pCRP-8D8 (Fig. 5b).

Each CRP subunit contains an intrasubunit disulfide bond. Reduced CRP migrates slower on SDS–PAGE than oxidized CRP, and both isoforms can thus be identified by separation of *N*-ethylmaleimide (NEM)-stabilized CRP under oxidizing conditions^27^. To determine the relative proportions of reduced and oxidized CRP in inflamed tissue, fresh lysates of burn wounds were treated with NEM and separated on SDS–PAGE. Burn wound lysates contained no detectable amounts of reduced CRP (Fig. 5c).

The intrasubunit disulfide bond can also be used to distinguish pentameric forms of CRP from mCRP, as low concentrations of DTT (dithiothreitol) are able to reduce mCRP but not pCRP (Supplementary Fig. 6A)^27^. We employed this assay to tissue lysates of human burn wounds, skeletal muscle tissue with ischemia/reperfusion injury (I/R injury) and carotid endarterectomy (CEA) specimens to determine the relative amounts of pentameric and monomeric CRP. Whereas control mCRP was efficiently reduced at 37 °C, only small amounts of tissue-deposited CRP could be reduced under the same conditions, indicating that the majority of CRP is in a pentameric form. If the samples were boiled, which induces pCRP dissociation, we observed nearly complete reduction of the intrasubunit disulfide bond (Fig. 5d). Quantification of western blots revealed that while burn wounds and CEA specimens contain significant amounts of mCRP, the majority of deposited CRP remained in a pentameric form (Fig. 5d). Detection of mCRP was not impaired by unknown components within the tissue lysates or precipitation of mCRP (Supplementary Fig. 6B,C). Since immunohistochemistry of burn wounds demonstrated that the neoepitope is exposed, the pentameric form in burn wounds and CEA specimens can be identified as pCRP*.

As our assay only indirectly identifies pCRP, we further analysed tissue lysates of burn wounds on SDS–PAGE with 1/20 of the commonly used SDS content. The low SDS concentrations preserve pCRP and allow separation of pCRP and mCRP^28^. Lysates showed a distinct band, migrating to the same height as control pCRP. This band was not visible in healthy skin tissue lysates and we did not observe a band that migrated at the height of mCRP (Fig. 5e). Gels were boiled in SDS–PAGE buffer before western transfer to enable uniform detection of CRP isoforms by anti-CRP-8 antibodies. These results confirm that CRP tissue deposits contain large amounts of pentameric CRP, mostly pCRP*.

As we observed a structural change from pCRP to pCRP* on the surface of liposomes and microvesicles, but no dissociation into mCRP by BN–PAGE (Fig. 4a,b), we also employed the red/ox-assay to these specimens. In addition to a higher sensitivity in mCRP detection (compare Fig. 5d to Fig. 5e), the assay analyses soluble CRP in the supernatant as well as the membrane-bound fractions. A conversion of pCRP to mCRP in the presence of liposomes or microvesicles was not found, confirming our BN–PAGE results (Fig. 5f). Liposomes did not prevent reduction of mCRP by DTT, as mCRP, which being added to liposomes could be effectively reduced (Fig. 5g).

### Microvesicle-bound pCRP* activates the complement system

**Figure 6:**
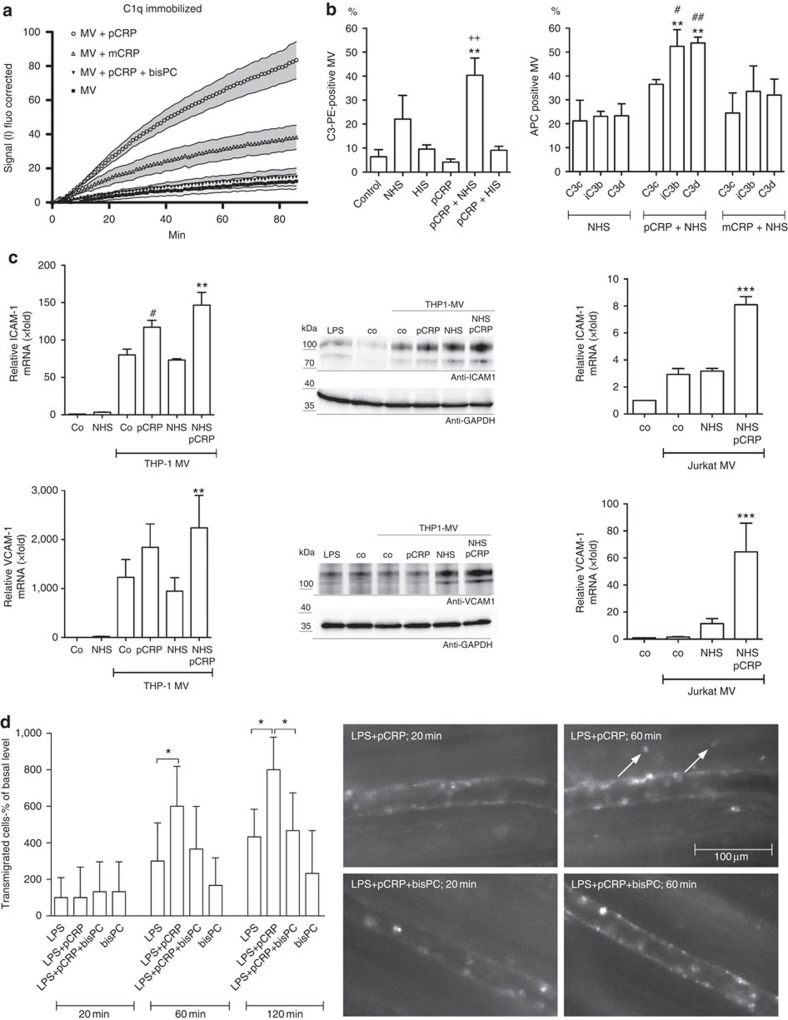
Proinflammatory effects of CRP and microvesicles. (**a**) Binding of CMFDA-labelled microvesicles (MV) to immobilized C1q was determined in the presence of pCRP, 1,6-bis-PC-pCRP and mCRP by repeated fluorescence measurements. 1,6-bis-PC is labelled as bisPC. Shaded areas display s.e.m. (*n*=3). (**b**) Binding of complement C3 to MV was assessed by flow cytometry with anti-C3b-PE antibodies (*n*=5), which recognizes C3, C3b and iC3b or three different C3 antibodies that recognize either the C3c part, the iC3b neoepitope or the C3d part, and a second APC-labelled antibody (*n*=3). pCRP* led to significantly increased C3 deposition that is present on the MV surface mainly in the form of iC3b. No deposition of complement C3 was observed in the absence of NHS or in the presence of heat-inactivated sera (HIS). Displayed are the means and s.e.m. *P* values were calculated with one-way analysis of variance (ANOVA). **P<0.01 to NHS, ^++^*P*<0.01 to HIS, ^#^*P*<0.05 to mCRP+NHS and ^##^*P*<0.01 to mCRP+NHS. (**c**) Expression of VCAM-1 and ICAM-1 by HUVECs was determined by qRT-PCR (quantitative real-time PCR) and western blot. pCRP+NHS significantly increased the expression of ICAM-1 and VCAM1 on HUVECs in the presence of cell-derived MV. Compared to THP-1 MV (*n*=4), Jurkat MV led only to a small increase in ICAM-1 and VCAM-1 expression (*n*=4). Displayed are the means and s.e.m. *P* values were calculated with one-way ANOVA. **P value <0.01 and ***P value <0.001 compared to NHS. ^#^*P* value <0.05 compared to THP-1-MV. Uncropped images of western blots are shown in Supplementary Fig. 14. (**d**) pCRP significantly increases the number of transmigrated leukocytes in LPS-induced inflammation in rat cremasteric postcapillary venules. This effect can be masked by preventing pCRP dissociation with 1,6-bis-PC. Transmigration of leukocytes was analysed by intravital microscopy under superfusion with LPS (25 ng ml^−1^)±intravenous injection of pCRP (25 μg ml^−1^) that had been preincubated with 1,6-bis-PC in some groups. After labelling with rhodamine 6G, the number of transmigrated leukocytes was quantified in a visual field of 200 μm in length and of 100 μm in width in the immediate vicinity of a postcapillary venule after 20, 60 and 120 min. Values are mean±s.e.m. of six rats per group; *P* values were calculated with one-way ANOVA, *P<0.05. Images shown are typical examples for postcapillary venules under LPS and pCRP in the presence and absence of 1,6-bis-PC after 20 and 60 min. Scale bar, 100 μm and applies to the whole panel.

Whereas circulating pCRP is a rather inert molecule, CRP bound to phospholipid membranes has been shown to activate the complement cascade^3^,^10^,^29^. We investigated whether pCRP* bound to microvesicles initiates the complement cascade. Binding of 5-chloromethylfluorescein diacetate (CMFDA)-labelled microvesicles to immobilized C1q was studied in the presence of mCRP and pCRP. Only few microvesicles bound to C1q in the absence of CRP. In contrast, both CRP isoforms led to increased binding, and pCRP was considerably more potent than mCRP (Fig. 6a). The interaction of microvesicles with C1q revealed a visually slower on-rate of microvesicles than the interaction with pCRP (Fig. 6a versus Fig. 4d). We also immobilized microvesicles and monitored the interaction with Alexa Fluor 488-labelled C1q. Only a small amount of C1q bound in the absence of pCRP, but we observed a similar increase and analogous binding characteristics in the presence of pCRP as that seen with immobilized C1q (Supplementary Fig. 7A).

We further studied complement activation on microvesicles by FACS with different complement component 3 (C3) antibodies directed against C3b (recognizes C3, C3b and iC3b), C3c (recognizes C3, C3b, iC3b and C3c), iC3b (iC3b neoepitope specific) and C3d (recognizes C3, C3b, iC3b and C3d). Microvesicles were incubated with normal human sera (NHS) and deposition of complement C3 determined by the C3b antibody. pCRP* significantly increased deposition of complement C3b on microvesicles, when compared to NHS only. In contrast, heat-inactivated sera and pCRP alone did not lead to C3b deposition (Fig. 6b). The subtype-specific antibodies confirmed that C3 is deposited on microvesicles even in the absence of CRP and further processed to iC3b. mCRP led only to a minor increase in C3 binding. However, in the presence of pCRP large amounts of C3, deposited mainly in the form of iC3b, were detected on the surface of microvesicles (Fig. 6b and Supplementary Fig. 7B).

### pCRP*–microvesicle complexes activate endothelial cells

Monocytic microvesicles activate endothelial cells in an IL-1β-dependent manner^30^. Incubation of human umbilical vein endothelial cells (HUVECs) with monocytic microvesicles leads to upregulation of adhesion molecules (for example, intercellular adhesion molecule-1 (ICAM-1) and vascular cell adhesion molecule 1 (VCAM-1)), which mediate recruitment of leukocytes to the site of tissue injury. Our study confirmed previous results that monocytic microvesicles lead to enhanced expression of ICAM-1 and VCAM-1 and that native pCRP does not increase their expression (Supplementary Fig. 7C).

We further assessed whether NHS and pCRP* influence the proinflammatory effects of microvesicles. Microvesicles from LPS-activated THP-1 cells were incubated with pCRP, NHS or pCRP+NHS, and their effects on VCAM-1 and ICAM-1 expression assessed by quantitative real-time PCR with reverse transcription and western blot. pCRP*-covered microvesicles led to an increase in ICAM-1 and VCAM-1 expression. This proinflammatory effect of pCRP* was even more pronounced in the presence of NHS, which led to a twofold increase of both adhesion molecules when compared to THP-1 cell-derived microvesicles only (Fig. 6c). The proinflammatory effects of pCRP* were not restricted to monocytic microvesicles and could also be observed with Jurkat cell-derived microvesicles. However, their potential to induce expression of cell adhesion molecules was about 20–30-fold lower (Fig. 6c).

To demonstrate the *in vivo* relevance of the proinflammatory pCRP* effects, we quantified leukocyte transmigration in the microcirculation of the rat cremaster muscle by intravital microscopy. In an established model for localized inflammation, the muscle tissue was superfused with low levels of LPS to induce cell activation and microvesicle release^4^. LPS triggered leukocyte evasion into the perivascular tissue and pCRP* significantly increased the number of transmigrated leukocytes. This proinflammatory effect of pCRP* could be inhibited by 1,6-bis-PC (Fig. 6d). Depletion of the complement system by pretreatment of rats with cobra venom factor abrogated most of the pCRP*-mediated proinflammatory effects, further highlighting the relevance of CRP*-mediated complement activation (Supplementary Fig. 8).

### Clearance of mCRP in circulation

Generating monomeric CRP by treating pCRP with 8 M urea in the presence of 10 mM EDTA for 2 h at 37 °C or by heating pCRP for 5 min at 95 °C in 0.1% SDS results in an unfolded protein similar in size, solubility, antigenicity and *in vitro* activity as the recombinant human mCRP C36A,C97A-double mutant protein^31^. The circular dichroism (CD) spectra for pCRP and mCRP (recombinant C36A,C97A mutant) demonstrate that these two CRP isoforms have different secondary structures (Supplementary Fig. 9A). The difference in the CD spectrum indicates that the mCRP protein is significantly more disordered than pCRP. This partially unfolded state might account for the rapid clearance of mCRP from circulation^32^,^33^. We examined whether mCRP and pCRP* influence uptake of CMFDA-labelled microvesicles in human macrophages. Only small amounts were engulfed in the absence of NHS, pCRP* and mCRP. NHS alone led to increased phagocytosis, which increased further in the presence of pCRP*. The effect of mCRP was even more pronounced, but contrary to pCRP*, NHS could not amplify its effects. If complement was inactivated by heat pretreatment, the sera failed to increase phagocytosis of microvesicles (Supplementary Fig 10).

### Model of pCRP* interaction with complement C1q

**Figure 7:**
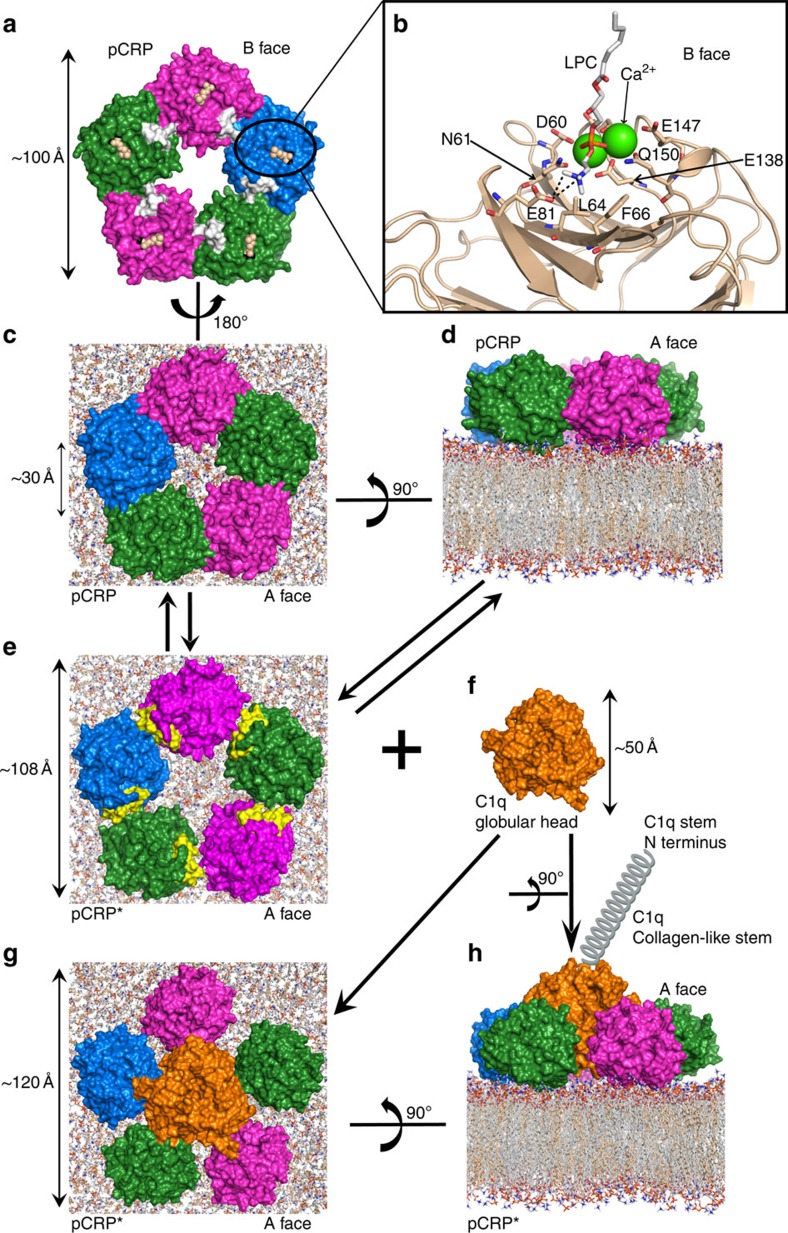
Model of the pCRP conformational rearrangement to pCRP* and interaction of pCRP* with complement C1q. (**a**) The individual subunits of human pCRP (PDB ID: 1B09; ref. 7) have been displayed as a molecular surface and coloured to highlight the subunit boundaries. View shown is the membrane binding B face of pCRP, with phosphocholine (cream spheres) and Ca^2+^ ions (black spheres) located in the ligand binding site on each subunit. The exposed region of the CHO-binding motif (residues 35–47) is coloured white in each subunit. The cross-sectional size of the pentamer is ∼100 Å (∼10 nm). (**b**) Close-up view of a phosphocholine head group from a microvesicle LPC molecule (light grey, orange, red, blue and white sticks) bound in the ligand binding site of one pCRP subunit (cream cartoon). Some of the residues lining the ligand binding site are labelled (using one letter amino-acid codes) and shown as sticks, the two Ca^2+^ ions are green spheres. Salt bridge interactions between the phosphocholine amine group and Glu81 (E81) are indicated by black dashed lines. (**c**) Interaction of pCRP with a model CHO:POPC bilayer (CHO shown as cream coloured sticks; POPC as light grey, orange, red, blue and white sticks). The ligand binding site on each pCRP subunit can bind to a phosphocholine head group of POPC as shown in (**b**) for an LPC molecule. View is from above, looking down onto the pCRP A face and showing the pentameric conformation. The diameter of the pentamer inner annular void is ∼30 Å (∼3 nm). (**d**) Side view showing the interaction between the B face of pCRP and the POPC phosphocholine head groups in the bilayer. The conversion of pCRP to pCRP* is a reversible process. (**e**) pCRP* in a pentameric conformation, same view as in (**c**). As the individual CRP subunits move apart, the neoepitope (residues 199–206, coloured yellow) is exposed and available for anti-mCRP-specific antibody (9C9 or 3H12) binding. The cross-sectional size of the pCRP* pentamer is ∼108 Å (∼10.8 nm). (**f**) The cross-section size of the complement C1q globular head group (PDB ID: 1PK6; ref. 47) is ∼50 Å (∼5 nm), this is the C1q domain that interacts with pCRP*. (**g**) and (**h**) The globular head of C1q inserts itself into the inner annular void of pCRP* forcing the subunits further apart. The interacting residues lie on the inner annular surface of the pCRP* pentamer and on the C-terminal surface of the C1q globular head group. The globular head group of C1q is physically unable to bind to pCRP. In (**h**) only one collagen-like fibre is shown (labelled as C1q collagen-like stem). We used part of a free art image for the coiled spring in (**h**) from www.vecteezy.com.

The crystal structure of native pCRP in complex with phosphocholine is shown in Fig. 7a. The phosphocholine (or phosphoethanolamine) ligand binding site is located in the groove of a β-sheet on the B face of pCRP. In Fig. 7b the phosphocholine head group of an LPC molecule has been docked into the ligand binding site. The phosphocholine moieties of the inhibitor 1,6-bis-PC have been shown to interact in a likewise manner^20^. When Ca^2+^ is not present (that is, in conditions of Ca^2+^ deficiency or addition of EDTA), residues 140–150 form a loop that projects away from the body of the CRP subunit exposing a normally hidden proteolysis site and the pCRP subunits dissociate as the individual subunit conformation unfolds^7^,^34^,^35^.

Activation of monocytic cells by LPS stimulation perturbs the cell membrane resulting in the exposure of PC, LPC, SM, PE and LPE lipid phosphocholine or phosphoethanolamine head groups, enabling pCRP to bind to the lipid surface (Fig. 1d,e). Only pCRP was detected on the surface of activated THP-1 cells or human monocytes (Fig. 4h). In contrast, both pCRP and pCRP* were detected on the surface of microvesicles (Fig. 4h,i). To demonstrate the interaction of pCRP with the surface of an LPS-activated THP-1 cell, or microvesicle, pCRP was docked to a model lipid bilayer containing CHO and 1-palmitoyl-2-deoylphosphatidylcholine (POPC) (Fig. 7c,d). The phosphocholine head groups of POPC interact in a similar manner to that depicted in Fig. 7b for LPC. There are distinct hydrophobic patches on the A and B faces of pCRP. These patches on the pCRP B face can interact with lipid rafts (that is, regions rich in CHO, Cer, PI, PS and SM) on the activated cell or microvesicle surface. A CHO binding motif (L/V-C-X(0-3)-YX(2-6)-R/K^36^) has been identified in CRP (residues 35–47). In pCRP, part of the CHO-binding motif is exposed on the surface (residues 42–47, coloured white in Fig. 7a) and is able to interact with CHO molecules in the lipid bilayer. PC, LPC, SM, PE and LPE lipid head groups also poke up into the pCRP subunit interface, where the phosphocholine (or phosphoethanolamine) amino group can break existing intersubunit interactions to form new interactions between the lipid head group and residues at the subunit interface.

The increased CHO and LPC content of microvesicles, compared to their cells of origin (Fig. 3a–d), increases the fluid nature (CHO and LPC) and the curvature (LPC) of the lipid bilayer further exposing the phosphocholine and phosphoethanolamine head groups of PC, LPC, SM, PE and LPE lipids^37^,^38^,^39^. Adjacent pCRP subunits (that is, CRP monomers) are held together by nine salt bridges, three hydrogen bonds and ten nonpolar interactions. The p*K*_a_ values of buried/partially buried aspartic and glutamic acid side-chains are significantly raised from their intrinsic values of 3.9 and 4.2, respectively. The experimentally determined average p*K*_a_ values for aspartic and glutamic acids in folded proteins are 3.5±1.2 (range 0.5–9.2) and 4.2±0.9 (range 2.1–8.8), respectively^40^. We have predicted the p*K*_a_ of the acidic residues on the intersubunit interface to be 8.4, 3.9, 4.6, 3.7, 6.1 and 4.0 for Asp155, Asp169, Glu42, Glu101, Glu108 and Glu197, respectively. The acidic pH observed at sites of inflammation and tissue injury (*in vitro* and *in vivo*, pH 3.6–5.7)^41^,^42^,^43^,^44^,^45^,^46^ will weaken the electrostatic intersubunit interactions (that is, salt bridges and hydrogen bonds) by protonating the acidic aspartic and glutamic acid side chains and the increased curvature of the surface will also assist to break the intersubunit cohesive forces (electrostatic and nonpolar interactions). The fluid motion of the microvesicle lipid bilayer provides a mechanical shear force and, coupled with the weakening of the pCRP intersubunit interactions, within 30 min on the surface pCRP starts to dissociate to pCRP* (ref. 13) as illustrated in Fig. 7e (refs 9,13, 41). As the CRP subunits move apart, they initially maintain an overall pentameric shape and the neoepitope (residues 199–206, coloured yellow in Fig. 7e and Supplementary Fig. 9B,C) become exposed^13^,^39^. The CRP subunits are only loosely associated with each other in pentameric pCRP*, with the electrostatic interaction distances being >4 Å. At this point, pCRP* has three main options: (1) to reform pCRP; (2) remain as pCRP* for interaction with complement C1q (or other interacting proteins) or (3) dissociate into mCRP (Supplementary Fig. 9B–D).

Complement C1q is a hexameric protein consisting of three separate chains^47^. The interaction between pCRP* and complement C1q is known to occur via the C1q globular heads (Fig. 7f–h). One of the collagen-like fibres and globular head is shown in Fig. 7h. Site-directed mutagenesis has identified residues on the C1q globular head involved in CRP binding^47^,^48^. The CRP residues determined by site-directed mutagenesis to interact with C1q^49^ all lie on the surface of the interior of the pentameric ring. The cross-sectional diameter of the C1q globular head is ∼50 Å (Fig. 7f), too large to fit into the interior void of pCRP (∼30 Å diameter; Fig. 7c); it is however, able to force the loosely associated pCRP* subunits further apart and thereby interact with the interior void of pCRP* (Fig. 7g,h). The C1q globular head was manually docked to pCRP* and the previously identified interacting residues lie on the C1q–pCRP* interface, consistent with our data showing that microvesicle-bound pCRP* recruits C1q and is responsible for activating the complement system (Fig. 6a,b).

The neoepitope (residues 199–206) lies at the pCRP intersubunit interface and is not available for anti-mCRP antibody 9C9 or 3H12 binding. Once the subunits dissociate to form pCRP* (Fig. 7e and Supplementary Fig. 9B,C), tryptophan fluorescence emission studies indicate that Trp205 flips out (red position in Supplementary Fig. 9C) and becomes solvent exposed. The increase in Trp205 solvent accessibility is consistent with our tryptophan fluorescence quenching data for pCRP bound to liposomes. The neoepitope is now accessible by the conformation specific anti-mCRP antibodies 9C9 and 3H12. In pCRP the C36–C97 intrasubunit disulfide bond (location is shown in Supplementary Fig. 9C) is protected from reduction, but once the neoepitope is exposed the disulfide bond is vulnerable to reducing conditions (for example, addition of DTT). Breaking of the intrasubunit disulfide bond results in the unfolding of the CRP subunit to produce the proinflammatory mCRP, that is, the conformation of CRP becomes disordered (Supplementary Fig. 9A), and all of the CHO-binding motif (residues 35–47) is now exposed. mCRP is eventually released and cleared from the circulation by the body's protein quality control mechanisms (Supplementary Figs 9D and 10).

## Discussion

**Figure 8:**
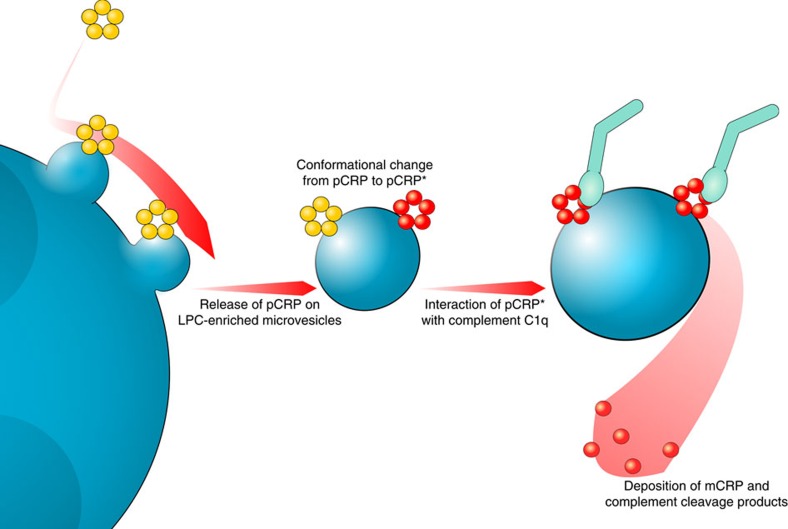
Model of CRP–microvesicle interaction. pCRP (yellow) binds to the plasma membrane of activated monocytes. It is subsequently released on membrane-derived microvesicles (blue). Microvesicle-associated pCRP is converted to pCRP* (red). pCRP* can activate the complement system by binding C1q (light green) or dissociate into individual mCRP subunits.

We have identified a novel, proinflammatory mechanism of CRP (Fig. 8). This is supported by the following findings: (1) circulating pCRP binds to the cell membrane of activated, but not resting, monocytes. Binding is observed *in vitro* and *in vivo*. (2) Stimulated cells release pCRP on LPC-enriched, proinflammatory microvesicles. Binding is specific, and can be inhibited by Ca^2+^ depletion and the CRP inhibitor 1,6-bis-PC. (3) Microvesicle-bound pCRP undergoes structural changes, leading to the expression of neoepitopes (pCRP*). pCRP* is the major isoform of CRP in human-inflamed tissue. (4) In contrast to pCRP, microvesicle-bound pCRP* exerts proinflammatory properties. It activates the complement system, amplifies the microvesicle-induced expression of cell adhesion molecules on endothelial cells and leads to increased recruitment of leukocytes to the site of tissue damage. (5) Molecular modelling confirms that activation of the complement system requires a structural change in the native protein, as complement factor C1q can be docked onto pCRP*, but not onto pCRP.

Binding of CRP to membrane phospholipids of apoptotic cells and Fcγ receptors of resting leukocytes has been studied in detail^8^,^11^,^50^. Our analysis additionally reveals activation-induced pCRP binding to monocytes, which explains why pCRP is not proinflammatory in the absence of existing tissue injury^3^,^6^,^29^.

Alterations of the LPC plasma membrane content, and membrane perturbations have been implicated in promoting pCRP binding to apoptotic cells^11^,^13^,^16^. We demonstrate binding to LPC-enriched microvesicles. pCRP attachment to microvesicles occurs via exposed phosphocholine or phosphoethanolamine head groups of phospholipids^24^,^37^. Blocking the binding sites in pCRP by 1,6-bis-PC, or removing the Ca^2+^ ions from their binding sites, precluded a stable interaction between pCRP and the microvesicles. This suggests an interaction with the B face of pCRP, leaving the A face accessible for C1q binding.

CRP bound to monocytes maintains the native pentameric structure of circulating pCRP. In contrast, CRP bound to microvesicles maintains pentameric symmetry but also reacts with neoepitope-specific antibodies (pCRP*). This is in line with previous reports, which show expression of neoepitopes on circulating microvesicles in patients with acute coronary syndrome^25^.

The concept that an enhanced membrane curvature is essential for neoepitope expression of CRP had been postulated previously^39^. This would explain why CRP does not elicit a proinflammatory response during the clearance of apoptotic bodies^50^. These vesicles are ∼1–5 μm in size and thus larger than microvesicles^51^. It is likely that pCRP* formation does not occur on apoptotic bodies because of their larger diameter, similar to intact cells. Thus, although pCRP binds to apoptotic fragments, the slight curvature of their membrane prevents the formation of pCRP*, and thus classical pathway activation. Still, some smaller apoptotic fragments might allow pCRP* formation and decoration of their surface with complement cleavage products. As pCRP* also recruits complement inhibitory factors, which lead to the formation of iC3b, only small amounts of anaphylatoxins are created. In addition, the inherent properties of the vesicles seem to be the major factors that determine the pCRP* effects. Apoptotic microvesicles elicit only a minimal inflammatory response, which is marginally augmented by pCRP* and complement. This is in contrast to the highly proinflammatory actions of monocytic microvesicles (Fig. 6).

Consistent with the published literature, it is likely that pCRP binding to multivalent ligands induces structural alterations in the pentamer to produce pCRP* (ref. 52). In addition, acidic pH, as occurring at sites of inflammation, can lead to exposure of buried epitopes^9^,^41^. pCRP* allows binding and activation of the complement system^6^,^13^,^52^. In reducing and/or Ca^2+^-depleted conditions often found at sites of tissue injury or inflammation, pCRP* further dissociates into individual unfolded subunits (mCRP), which might contribute to the clearance of cell debris and activation of phagocytic cells^4^,^16^. As pCRP* is abundantly found in injured tissues, it might represent the inflammatory active isoform that is later cleared as mCRP. Previous data showing deposition of mCRP in inflamed tissue might therefore reflect a snapshot of the end point of the proinflammatory activation cascade that has taken place.

This is consistent with our data, which show that pCRP* is more potent in binding C1q and activating the classical complement pathway than mCRP. Still, mCRP maintains partial complement-activating activity and is a strong opsonin, facilitating efficient uptake of mCRP-decorated vesicles. These properties might explain the short half-life of mCRP in the circulation. Rather than reflecting a conflict with previous data, we hypothesize that the involvement of both isoforms represents different time points in the proinflammatory cascade (Fig. 8)^4^,^32^. The data presented here confirm previous studies that the proinflammatory properties of CRP are regulated by conformational changes^4^,^13^.

Besides the above-mentioned proinflammatory effects, anti-inflammatory properties have been attributed to pCRP. pCRP has been shown to protect mice against endotoxin shock^53^,^54^. Although the mechanisms of this controversy are not entirely clear, it is possible that the pro- or anti-inflammatory properties of pCRP depend on the extent of pre-existing inflammation. The intravital microscopy model that we adopted (Fig. 6d) uses only very low concentrations of locally applied LPS solution (25 ng ml^−1^). In contrast, mice in the above-mentioned endotoxin shock model were challenged with much higher doses of LPS (15,000–27,000 ng per mg body weight). Thus, pCRP might exert a dual role; it might aggravate low-level inflammatory responses as seen in sterile tissue injury (or low-level LPS administration), and also reduce excessive inflammation, as occurring in endotoxin shock.

Still, the inflammatory response in an endotoxin shock is a complex interplay of pro- and anti-inflammatory mechanisms. The compensatory anti-inflammatory response syndrome, which leads to a systemic deactivation of the immune system, can lead to failure of proinflammatory defense mechanisms against infectious organisms^55^. In this scenario, the ability of CRP to promote a proinflammatory response would be beneficial.

Taken together, our results unravel a mechanism of how CRP exerts its proinflammatory effects in damaged tissue on a molecular level. The small-molecule inhibitor 1,6-bis-PC blocked binding of pCRP to microvesicles, inhibited pCRP-dependent C1q attachment and abrogated CRP-mediated leukocyte recruitment. Small-molecule CRP inhibitors may therefore be a promising approach for the treatment of inflammatory conditions such as acute myocardial infarction and stroke^2^,^4^,^20^. However, the limited potency and pharmacokinetic properties of 1,6-bis-PC currently restricts its use as a therapeutic agent. Our data warrant further research towards the development of more potent and orally bioavailable ‘CRP inhibitors'. The potential benefits for a broad scope of inflammatory diseases, as well as various I/R injuries, make this a highly attractive novel drug class.

## Methods

### CRP preparations

Human pCRP was purchased from Calbiochem (Nottingham, UK) and thoroughly dialysed against Dulbecco's PBS (D-PBS) supplemented with 2 mM Ca^2+^. Structural and functional integrity of pCRP was verified by native gel electrophoresis and liposome binding studies. Commercial pCRP migrated at the same height as serum CRP, showed no contaminations with mCRP and bound in a Ca^2+^-dependent manner to LPC-containing liposomes (Supplementary Fig. 11A,B). mCRP was generated by treating pCRP with 8 M urea in the presence of 10 mM EDTA for 2 h at 37 °C or by heating for 5 min at 95 °C in 0.1% SDS. mCRP was thoroughly dialysed in low salt phosphate buffer (10 mM Na_2_HPO_4_, 10 mM NaH_2_PO_4_, and 15 mM NaCl, pH 7.4)^14^,^56^. Recombinant human C36A,C97A mCRP was purified from inclusion bodies using reversible anhydride modification^57^. To detect any bacterial contamination of CRP preparations, all reagents were tested for LPS contamination with a Limulus assay (Sigma-Aldrich, Taufkirchen, Germany) and it was found to be below the detection limit (0.125 U ml^−1^ or 0.01 ng ml^−1^ LPS).

### Antibodies

Anti-mCRP-9C9 and anti-pCRP-8D8 antibodies were purified from hybridoma culture supernatants^58^. Anti-CRP antibody clone CRP-8, mouse IgG FITC-conjugated F(ab′)_2_ and human IgG-purified immunoglobulins were purchased from Sigma-Aldrich. CD16-FITC, CD32-FITC, CD64-FITC, CD14-PE, CD68-PE, CD80-FITC, CD163-FITC and respective control antibodies were from Miltenyi Biotec (Bergisch Gladbach, Germany). Anti-ICAM-1 (#4915) and NF-κB pathway antibodies (NF-κB Pathway Sampler Kit; #9936) were from Cell Signaling Technology (Danvers, MA, USA). Anti-VCAM-1-horseradish peroxidase (anti-VCAM-1-HRP) (E-10) and anti-GAPDH-HRP (0411) were purchased from Santa Cruz Biotechnology (Dallas, TX, USA). Complement C3-PE antibody (clone 6C9), which reacts with human C3 as well as the breakdown products C3b and iC3b, was purchased from LifeSpan BioSciences (Seattle, WA, USA) and Alexa Fluor 488 Annexin V from Life Technologies (Carlsbad, CA, USA). APC goat anti-mouse IgG was from BD Biosciences (San Diego, CA, USA). Anti-iC3b (neoepitope), anti-C3c (recognizes C3, C3b, iC3b, C3c) and anti-C3d (recognizes C3, C3b, iC3b, C3d) antibodies were purchased from Quidel (San Diego, CA, USA).

### Cells

Monocytes were isolated from peripheral venous blood of healthy human volunteers by density gradient centrifugation. All volunteers gave informed consent before enrolment and approval from the relevant ethics committee was obtained (ethics review boards, University of Freiburg, Freiburg, Germany; 343/13). Monocytes, THP-1 acute monocytic leukemia cells (source: DSMZ, Braunschweig, Germany) and Jurkat T-cell leukemia cells (source: Center for Chronic Immunodeficiency, Freiburg, Germany) were cultured in 90% RPMI-1640 media, 10% FCS, 2 mM L-glutamine with 50 U ml^−1^ penicillin and 50 μg ml^−1^ streptomycin. Identity of cell lines was confirmed by *Multiplex human Cell line Authentication Test* (Multiplexion, Heidelberg, Germany). HUVECs were bought from PromoCell (Heidelberg, Germany) and cultured in Endothelial Cell Basal Medium with SupplementMix (PromoCell), 10% FCS, 50 U ml^−1^ penicillin and 50 μg ml^−1^ streptomycin.

### Intravital microscopy of rat cremaster muscle

Animal studies were in compliance with ethical regulations and approved by the institutional animal care committee (35-9185.81/G-10/114) at the University of Freiburg. Male Wistar rats (Charles River, Sulzfeld, Germany) weighing 120–180 *g* (age: 5–6 weeks) were anaesthetized with 1.5 vol% isoflurane (Abbott, Wiesbaden, Germany). After tracheotomy, respiration was volume controlled (frequency, 35–45 breaths per min; tidal volume, 4.5–5 ml; FiO_2_, 0.35–0.50; Servo Ventilator 900C, Maquet, Rastatt, Germany). The right carotid artery and the left jugular vein were cannulated for continuous monitoring of mean arterial pressure, heart rate, blood gases and acid/base status. The right cremaster muscle was externalized over a heated aluminium platform and prepared for visualization of the microcirculation as previously described^59^. After coverage with a square coverslip, the cremaster muscle was continuously superfused at 1 ml min^−1^ with PBS-Ca^2+^-Mg^2+^ warmed to 37 °C. The tissue was then allowed to stabilize for 20 min. Digital intravital epifluorescence microscopy of the cremaster muscle was performed using a modified Zeiss microscope (Axio Scope A-1 MAT) equipped with a water immersion objective lens (× 20/1.0) and a Colibry 2 system for fluorescence records (Zeiss). Observations were recorded by a high-resolution digital camera (AxioCam MRm Rev. 3 FireWire, Zeiss). The cremaster muscle was then constantly superfused with indicated concentrations of LPS for 60 min. After 20 min, an L-pCRP solution bolus (25 μg ml^−1^ serum concentration) was injected intravenously. pCRP was fluorescently labelled with Alexa Fluor 594-labelled reactive dye that has a succinimidyl ester moiety reacting with primary amines of pCRP to form stable dye-protein conjugates (L-pCRP). Purification of the labelled protein and determination of the degree of labelling were performed according to the manufacturer's protocol (Protein Labelling Kit; Life Technologies). For decameric stabilization, 1 mg ml^−1^ pCRP solution was incubated with 1,6-bis-PC in 1:100 molar ratio for 30 min at 37 °C. Weight-dependent rat serum volume was calculated and CRP serum levels were verified by a particle-enhanced immunoturbidimetric assay (Modular Analytics; Roche Diagnostics, Basel, Switzerland). The fluorescence signal was measured at 15 min intervals.

Leukocyte endothelial interactions and leukocyte transmigration in the cremaster muscle were observed by intravital microscopy as previously described^4^. Transmigrated leukocytes were measured after indicated times for 20 s in an area of 20,000 μm^2^ (200 μm length along the postcapillary venule and 100 μm depth into the cremasteric muscle tissue). To investigate the role of the complement system in CRP-mediated inflammation *in vivo*, rats were treated with cobra venom factor (250 U kg^−1^ body weight via intraperitoneal injection) 24 h before intravital imaging. We used the AxioVision software (Zeiss, Jena, Germany) to measure transmigration of rhodamine 6G-labelled leukocytes.

### Sample collection and preparation of tissue lysates

All patient recruitment was performed following approval from the relevant institutional human ethics committees (ethics review board, Freiburg, Germany, projects 24/13 and 67/08) in accordance with the Declaration of Helsinki. All patients gave informed consent before enrolment. Burned skin was collected from patients with deep second-degree to full-thickness burn wounds, who needed surgical excision on days 4–8 following injury. Further patient characteristics can be found in Supplementary Table 1. Healthy control skin was taken from excess material of abdominoplasty procedures. Atherosclerotic plaques were taken from patients needing carotid endarterectomy and human skeletal muscle biopsies were taken from patients needing soft tissue reconstruction by means of free muscle transfer as previously described^4^. Tissue specimens were directly frozen in liquid nitrogen and stored at −80 °C.

Frozen specimens were cut into small cubes and incubated in lysis buffer (25 mmol l^−1^ Tris-HCl (pH 7.4), 150 mmol l^−1^ NaCl, 2 mmol l^−1^ CaCl_2_, 0.5% Triton X-100, 1 mM orthovanadate, 10 μg ml^−1^ leupeptine, 10 μg ml^−1^ aprotinin and 1 mM phenylmethylsulfonyl fluoride) for 30 min on ice. Tissue was homogenized with an Ultra-Turrax disperser from IKA (Staufen, Germany) and centrifuged at 17,000 *g* for 10 min at 4 °C to remove insoluble components. To assess the oxidation state of the intrasubunit disulfide bond, 100 mmol l^−1^ NEM (Sigma-Aldrich) was added to the lysis buffer to stabilize reduced CRP monomers. Protein concentrations were determined with a BCA Protein Assay Kit from Pierce (Rockford, IL, USA).

### Histology of human skin

Staining was performed on native tissue sections^16^. After incubation with the primary antibody (clone CRP-8 1:200; anti-mCRP 9C9 1:20; anti-pCRP 8D8 1:20) for 1 h at room temperature, slides were incubated with the undiluted HRP-labelled anti-mouse antibody for 30 min. Reaction products were stained with HistoGreen Substrate Kit for peroxidase from Linaris (Dossenheim, Germany) resulting in a green reaction product.

### SDS–PAGE, BN–PAGE and western blot

For SDS–PAGE, tissue lysates were precipitated with an equal volume of 10% trichloroacetic acid on ice. Protein pellets were denatured at 95 °C for 5 min in SDS loading dye (with or without DTT) and separated on 15% SDS–polyacrylamide gels. Native electrophoresis was performed by BN–PAGE as previously described^60^. Proteins were separated on 4–16% native PAGE Bis-Tris gels from Life Technologies (Darmstadt, Germany). Gels were subsequently incubated for 5 min at 95 °C in 1x gel buffer containing 10 mM DTT and 1% SDS. After western blot, PVDF membranes were blocked in 5% milk powder in TBS-T and incubated with clone CRP-8 antibodies (1:500), followed by a HRP-conjugated anti-mouse antibody (1:5,000) in 1% bovine serum albumin (BSA) TBS-T. For western blot, we uniformly used anti-CRP-8 antibodies. Native gels (or 1/20 SDS–PAGE gels) were always boiled before western transfer, which induces pCRP dissociation and thus enables a uniform transfer and detection of all CRP isoforms by anti-CRP-8 antibodies. These experiments are thus independent of conformation specificity of antibodies. Respective pCRP or mCRP controls confirm the feasibility of this approach. ECL Western blotting Detection Reagents (GE Healthcare, Buckinghamshire, UK) were used to visualize protein bands in a Fusion Fx7 chamber (Peqlab, Erlangen, Germany). Band intensity was determined with ImageQuant 5.2 (Molecular Dynamics, Sunnyvale, CA USA) and CRP binding was calculated relative to an input control.

### NF-κB pathway screen

THP-1 cells (1 × 10^6^ per ml) were incubated with LPS (10 μg ml^−1^) for indicated time periods and the reaction stopped by putting the cells on ice. Cells were washed once in ice-cold PBS (520 *g* for 5 min) and lysed in RIPA buffer with protease/phosphatase inhibitors (Cell Signalling Technology). The lysate was briefly sonicated and centrifuged at 14,000 *g* for 10 min. Supernatant fluids were collected and the protein concentration determined by BCA assay. Equal amounts of protein were mixed with 2 × SDS loading buffer and separated on 12.5% SDS–PAGE. Detection occurred after western blotting as described above; however, blocking of the PVDF membrane was performed with 1% BSA TBS-T instead of milk powder due to the subsequent use of phosphorylation-specific antibodies.

### Determination of CRP isoforms in tissue

pCRP and monomeric CRP were distinguished by two different methods. Electrophoresis on polyacrylamide gels containing reduced amounts of SDS can separate pCRP (pCRP*) from mCRP as described by Taylor and van den Berg^28^. The low SDS content preserves the pentameric structure of pCRP/pCRP* and allows its separation from mCRP due to its slower migration velocity on polyacrylamide gels. Tissue lysates were prepared as described above, loaded in native buffer and separated on 8–13% polyacrylamide gels with 1/20 SDS content.

A method to differentiate between pCRP/pCRP* and mCRP has recently been published by Wang *et al*.^27^ Each CRP subunit contains a disulfide bond, which is accessible to low concentrations of DTT in mCRP but not in pCRP/pCRP*. As reduced CRP subunits migrate slower on SDS–PAGE than oxidized subunits, the accessibility of the intrasubunit disulfide bond can be used to identify mCRP. Tissue lysates, control pCRP and mCRP were incubated in 25 mmol l^−1^ Tris-HCl (pH 7.4), 150 mmol l^−1^ NaCl and 2 mmol l^−1^ CaCl_2_ in the presence and absence of 10 mM DTT at 37 °C for 2 h. The reaction was stopped by adding 100 mmol l^−1^ NEM for 15 min at 25 °C, which stabilizes reduced disulfide bonds during gel electrophoresis. Samples were precipitated with an equal volume of 10% trichloroacetic acid at 4 °C. The pellet was washed once in ice-cold acetone, resuspended in non-reducing SDS-loading dye and separated on a 15% non-reducing SDS–PAGE.

### Microvesicle isolation

Microvesicles originate from cell membranes and exhibit a size of ∼100–1,000 nm. THP-1 microvesicles were purified by differential centrifugation according to a recently published protocol with slight modifications^30^. Briefly, THP-1 cells (1 × 10^7^ per ml) were treated with 10 μg ml^−1^ LPS (*Escherichia coli* serotype O127:B8, Sigma-Aldrich) and Jurkat cells with 1 μM staurosporine (Sigma Aldrich) for 5 h in FCS-free media. Cells were centrifuged at 500 *g* for 5 min at room temperature. The supernatant was centrifuged at 1,500 *g* for 15 min. The supernatant containing microvesicles was centrifuged at 16,000 *g* for 60 min at 4 °C to pellet the microvesicles (P 16,000 *g*). Platelet microvesicles were purified as recently described^61^. Human polymorphonuclear leukocytes (PMNLs) were purified as described^62^. PMNLs (1 × 10^7^ per ml) were treated with 10 nM PMA or 1 μM fMLP (*N*-formylmethionyl-leucyl-phenylalanine) for 20 min at 37 °C. Cells were double centrifuged at 4,000 *g* for 20 min at room temperature. The supernatant containing microvesicles was processed as described above for THP-1 microvesicles. Human monocytes were isolated from peripheral blood mononuclear cells (PBMCs) by negative selection (EasySep Human Monocyte Enrichment Kit; Stemcell Technologies, Köln, Germany). Purified monocytes (1 × 10^7^ per ml) were incubated with 10 μg ml^−1^ LPS for 2 h. Microvesicles were purified as described for THP-1 cells.

All microvesicle preparations were washed twice with PBS supplemented with 2 mM Ca^2+^ and stored at −80 °C until use. The amount of microvesicles was quantified by the total protein concentration determined by a BCA Protein Assay Kit. Microvesicles were highly pure, as analysed by flow cytometry (Supplementary Fig. 11C). Smaller vesicles, including exosomes (50–100 nm), were isolated from the 16,000 *g* supernatant by further ultracentrifugation (TLA 100.2 rotor; Beckman Coulter) at 100,000 *g* for 60 min at 4 °C (P 100,000 *g*), and of the 100,000 *g* supernatant (SN 100,000 *g*) at 400,000 *g* for 60 min at 4 °C (P 400,000 *g*).

### Flow cytometry

Human monocytes or THP-1 cells (2 × 10^6^ per ml) were incubated with 100 μg ml^−1^ pCRP for indicated time periods in FACS buffer (PBS-Ca-Mg, 1% BSA) at 37 °C. Cells were pelleted (520 *g*, 5 min) and the reaction stopped by resuspending the cells in ice-cold FACS buffer with 0.1% sodium azide. Cells were stained with anti-pCRP-8D8 (1:50) or anti-mCRP-9C9 (1:50) antibodies for 30 min. After washing, cells were incubated with 500 nM 4,6-diamidino-2-phenylindole (DAPI) (Sigma-Aldrich) and a FITC-labelled anti-mouse F(ab)_2_ (1:200). Cells were again washed and subsequently analysed by flow cytometry (BD LSR Fortessa Cell Analyzer). Only DAPI-negative cells were analysed to exclude non-viable cells. Microvesicles were stained as described for cells; however, each centrifugation step occurred at 16,000 *g* for 30 min. The microvesicle gate was defined by size, using 0.88, 1.1 and 1.34 μm beads (Spherotech, Lake Forest, USA), and the forward scatter threshold set to 400 to exclude gating exosomes.

### Quantification of CRP on microvesicles from ST elevation myocardial infarction patients

All patient recruitment was performed following approval from the relevant institutional human ethics committees (ethics review boards, Alfred Medical Research and Education Precinct Project 516/13) in accordance with the Declaration of Helsinki. All patients gave informed consent before enrolment. We recruited patients who presented with acute ST elevation myocardial infarction and underwent primary percutaneous intervention of a coronary artery. The clinical inclusion criteria were age between 18 and 80 years, coronary artery disease status established by angiography and willing and able to provide informed consent. To enable sufficient time for serum pCRP to rise and enhance the probability of pCRP*/mCRP detection, all patients were enrolled 24 to 48 h after angiography. Healthy volunteers were recruited as controls.

Blood samples were collected using vacationer tubes with heparin as the anticoagulant. The blood was centrifuged at 3,000 *g* for 15 min within 30 min of phlebotomy. The plasma was carefully collected and centrifuged at 12,000 *g* for 2 min. About 90% of the supernatant (platelet-free plasma) was carefully collected into fresh tubes and snap frozen in liquid nitrogen before storing them at −80 °C. Frozen samples were thawed in a 37 °C water bath for 5 min, vortexed and then centrifuged at 3,000 *g* for 15 min. Once again ∼90% of the supernatant (platelet-free plasma) was carefully collected into fresh tubes and centrifuged at 14,000 *g* for 60 min. The supernatant was discarded and the remaining pellets (microvesicles) were reconstituted with PBS. The buffer was filtered twice through a 0.2 μm membrane filter before use. Twenty microliters of microvesicles were incubated with either 5 μl of anti-mCRP-9C9 or 5 μl of anti-pCRP-8D8. A measure of 0.2 μl of anti-mouse IgG-FITC antibody (Sigma, USA) was used as secondary antibody. Dual staining was performed by adding PE-conjugated anti-human CD41, CD11b or CD62P into the sample. Control samples included isotype controls and single stain samples. All flow cytometry analyses were performed using the LSRFortessa (BD Bioscience, USA) and the system is set to collection of all events for 90 s.

### Complement deposition on microvesicles

THP-1 microvesicles (25 μg ml^−1^) were incubated with or without pCRP (40 μg ml^−1^) for 30 min at 37 °C in 25 mmol l^−1^ Tris-HCl (pH 7.4), 150 mmol l^−1^ NaCl, 2 mmol l^−1^ CaCl_2_. Ten per cent NHS or HIS (56 °C, 45 min) was added for a further 15 min before microvesicles were pelleted for 30 min at 16,000 *g* at 4 °C. Microvesicles were resuspended in Annexin V-binding buffer and stained with C3-PE (1:200) and Alexa Fluor 488 Annexin V (1:200) for 30 min at 20 °C. Subsequently, microvesicles were pelleted (16,000 *g*, 30 min, 4 °C), resuspended in Annexin V-binding buffer and analysed by two-colour flow cytometry.

For C3c, iC3b and C3d analysis, THP-1 cells were labelled with CMFDA (Life Technologies) according to the manufacturer's instructions before microvesicle release and purification. CMFDA-labelled microvesicles were treated as described above. Staining was performed with the respective C3 antibodies (1:200) and a second APC anti-mouse IgG (1:200). Microvesicle gates were defined as described under ‘flow cytometry'.

### Quantification of microvesicles by flow cytometry

THP-1 cells were washed twice at 530 *g* for 5 min in PBS to remove contaminating microvesicles from the media and finally resuspended in Annexin V-binding buffer (BD Biosciences) at a concentration of 1 × 10^6^ per ml. Cells were transferred in FACS tubes and Alexa Fluor 488 Annexin V (1:100) added. LPS (10 μg ml^−1^) and pCRP (40 μg ml^−1^) were added and cells were left at room temperature. Released microvesicles were analysed as described in ‘flow cytometry'. Microvesicles were defined as Annexin V-positive particles <1.1 μm.

### Microvesicle release by M1/M2 macrophages

Human PBMCs were differentiated into M1 or M2 macrophages with PromoCell Macrophage Generation Media (PromoCell GmbH, Heidelberg, Germany) according to the manufacturer's instructions. Differentiation into M1 or M2 macrophages was verified by flow cytometry with CD68-PE/CD80-FITC (M1 macrophages) and CD68-PE/CD163-FITC (M2 macrophages) according to the manufacturer's recommendations. Target antibodies and control antibodies were obtained from Miltenyi Biotec. Cells were washed twice with PBS and subsequently incubated with PMA (50 ng ml^−1^), MPLA (1 μg ml^−1^), LPS (1 μg ml^−1^) or PBS in Annexin-binding buffer supplemented with 10% FCS. The supernatant was collected at indicated time points and centrifuged at 1,500 *g* for 15 min to pellet contaminating cells and cell debris. Microvesicles were stained with AnnexinV-FITC (1:200; BD Pharmingen) and counted by flow cytometry^23^. True count beads (BD Biosciences, San Jose, CA, USA) were used for absolute quantification.

### Fluorescence microscopy

Cells were stained as described in ‘flow cytometry' and immobilized on adhesion microscope slides (Paul Marienfeld, Laura-Königshofen, Germany) according to the manufacturer's protocol. Cells were fixed with 2% formaldehyde, permeabilized with 0.05% PBS-Tween and mounted with Vectrashield Mounting Media with DAPI (Vector Laboratories, Burlingame, CA, USA). Image acquisition was performed by confocal microscopy (Leica TCS SP2 AOBS; Leica Microsystems, Wetzlar, Germany) and processed with the Leica Confocal Software. Magnification of the objective was × 63, scanner speed set to 800 Hz and the pinhole diameter was 1 airy unit. FITC was excited at 488 nm and DAPI at 405 nm.

### Liposome- and microvesicle-binding studies

Lipids were purchased from Avanti Polar Lipids (Alabaster, AL, USA). Large multilamellar liposomes were prepared as previously described^63^. Liposomes had the following compositions: PC liposomes (40% CHO, 60% PC); PC/LPC liposomes (40% CHO, 40% PC, 20% LPC); P 16,000 *g* liposomes (50% CHO, 7% Cer, 10% SM, 4% PS, 1% LPE, 1% LPC, 27% PC); SN 100,000 *g* liposomes (6% Cer, 11% SM, 6% LPC, 5% LPE, 3.5% PS, 68.5% PC).

pCRP of 40 μg ml^−1^ was incubated with 200 μg ml^−1^ liposomes or 200 μg ml^−1^ microvesicles (P 16,000 *g*) at 37 °C for 1 h in 25 mM Tris-HCl (pH 7.4), 150 mM NaCl, 2 mM CaCl_2_ and 1 mg ml albumin. Ten millimoles of EDTA was added to inhibit Ca^2+^-dependent CRP binding. A specific small-molecule inhibitor of CRP, 1,6-bis-PC, was synthesized by Syngene International (Bangalore, India) and used at a concentration of 10 mM^20^. Liposomes and microvesicles were subsequently pelleted for 30 min at 110,000 *g* at 4 °C. The pellet was resuspended in lysis buffer and separated on 4–16% Bis-Tris gels as described under ‘BN–PAGE'. pCRP and mCRP controls were directly dissolved in lysis buffer.

### Lipididomics

To determine the lipid profile of whole THP-1 cells, 1 × 10^6^ per ml cells were stimulated with LPS (10 μg ml) in RPMI-1640 media supplemented with 10% FCS for indicated time periods. Cells were then washed in PBS, resuspended in 20 mM Tris-HCl (pH 7.4), 500 mM NaCl and lysed with a tip sonicator. The protein concentration of the lysate was determined and adjusted to 1 mg per ml. THP-1 microvesicles were purified from FCS-free media, as FCS contains microvesicles and high concentrations of LPC. Purification was performed as described in ‘microvesicle isolation'; however, cells were only stimulated for 2 h and the cell concentration was adjusted to 2 × 10^6^ per ml. Platelet microvesicles were purified as described above. Pelleted microvesicles were washed in PBS, and finally resuspended in 20 mM Tris-HCl (pH 7.4) and 500 mM NaCl. The supernatant (200 μl) of the individual centrifugation steps was lyophilized and resuspended in 10 μl of 20 mM Tris-HCl (pH 7.4) and 500 mM NaCl.

Samples underwent total lipid extraction, using a single-phase chloroform/methanol (2:1) technique as described previously^64^. A 10 μl aliquot of cell lysate, microvesicles or lyophilized cell supernatant was combined with 200 μl CHCl_3_/MeOH (2:1) and 15 μl of internal standard mix and then briefly vortexed. Samples were mixed (rotary mixer, 10 min), sonicated (water bath, 30 min) and then allowed to stand (20 min) at room temperature. Samples were centrifuged (16,000 *g*, 10 min) and the supernatant was dried under a stream of nitrogen at 40 °C. The extracted lipids were then resuspended in 50 μl H_2_O saturated BuOH with sonication (10 min), followed by 50 μl of 10 mM NH_4_COOH in MeOH. Extracts were centrifuged (3,350 *g*, 5 min) and the supernatant transferred into 0.2 ml glass vials with Teflon insert caps. Mass spectrometric analysis was performed using 5 μl injections of the lipid extracts. Lipid analysis was performed by liquid chromatography, electrospray ionization–tandem mass spectrometry using an Agilent 1,200 liquid chromatography system combined with an Applied Biosystems API 4000 Q/TRAP mass spectrometer with a turbo ion-spray source (350 °C) and the Analyst 1.5 data system. Quantification of lipids was based on signal intensity relative to the corresponding internal standard as described previously^65^. Results were then given in pmol ml^−1^ or pmol mg^−1^. For lyophilized samples, background values of buffer or media were subtracted. Values displayed for each lipid class were calculated as the sum of each individual species within the glass.

### ICAM-1 and VCAM-1 expression of HUVECs

HUVECs were cultured in 6-well plates. Confluent HUVECs were serum-starved for 4 h and then incubated with microvesicles (25 μg ml^−1^ of total protein), NHS (10%), pCRP (40 μg ml^−1^), mCRP (40 μg ml^−1^) or LPS (10 μg ml^−1^) for 2 h at 37 °C in serum-free endothelial cell growth medium. Cells were then lysed in RLT buffer (Qiagen, Venlo, The Netherlands), and RNA was isolated using the RNeasy Mini Kit (Qiagen). Total RNA was quantified by NanoDrop (Thermo Scientific, Waltham, MA, USA), and digested with DNase I (Life Technologies). Total RNA of 0.5 μg was reverse transcribed into cDNA using the AffinityScript cDNA Synthesis Kit (Agilent Technologies, Santa Clara, CA, USA) according to the manufacturer's instruction. mRNA levels were analysed by real-time PCR using ABsolute QPCR ROX Mix (Thermo Scientific). Primers and probes for human ICAM-1 (Hs00164932_m1), VCAM-1 (Hs01003372_m1) and RNA18S5 (Hs03928985_g1) were purchased from Applied Biosystems (Life Technologies). Amplification of RNA18S5 was used for normalization. All experiments are expressed as relative expression normalized to untreated HUVECs according to the 2^−ΔCT^ method^66^. For western blots cells were treated as described above. However, incubation with the respective microvesicle preparation was conducted for 4 h. Cells were directly lysed in ice-cold RIPA buffer, processes as described under ‘NF-κB pathway screen' and stained with the respective antibodies against ICAM-1 (1:1,000), VCAM-1 (1:200) and GAPDH (1:200).

### Real-time kinetic measurements

Kinetic measurements were obtained by direct detection of fluorescently labelled compounds. C1q and pCRP were labelled with Alexa Fluor 488 (Life Technologies) and microvesicles with CMFDA, as described above. To monitor the interaction of fluorescently labelled pCRP/C1q with unlabelled microvesicles, purified THP-1 microvesicles (0.5 mg ml^−1^) were spotted on a nitrocellulose membrane, left to dry for 2 min and subsequently blocked with 5% BSA in TBS-T. After washing, labelled pCRP/C1q was added at the indicated concentrations and repeated fluorescence measurements of microvesicle-associated pCRP/C1q and respective reference area values were obtained by a LigandTracer Green with a FITC-compatible detector and analysed using Trace Drawer 1.6 (Ridgeview Instruments AB, Vänge, Sweden). The differential signal (microvesicle area minus reference area) becomes a background-corrected measure for the amount of pCRP/C1q attached to the microvesicles. When indicated, 50 mM EDTA was added to the Alexa Fluor 488-labelled pCRP solution. To determine the binding of CMFDA-labelled microvesicles to unlabelled immobilized C1q, 60 μg ml^−1^ human C1q (Merck Chemicals GmbH, Darmstadt, Germany) was spotted on a nitrocellulose membrane. If indicated, microvesicles (0.5 mg ml^−1^) were preincubated for 2 h at 37 °C with pCRP (100 μg ml^−1^), mCRP (100 μg ml^−1^) or 1,6-bis-PC–pCRP complexes (100:1 molar ratio 1,6-bis-PC–pCRP) before they were added at a final concentration of 20 μg ml^−1^. Repeated fluorescence measurements were obtained as described above.

### Modelling

All modelling and geometry optimization was performed using SYBYL-X 2.1 (Certara LP, http://www.tripos.com). LPC molecules were constructed using standard bond lengths and bond angles. The crystal structure of pCRP in complex with phosphocholine (PDB ID: 1B09; ref. 7) was manually docked to either a model 1:2 CHO:POPC lipid bilayer^67^ or LPC molecules by aligning the phosphocholine head groups of POPC or LPC to the crystal bound phosphocholine molecules. The crystal-bound phosphocholine ligands were then removed to create either the LPC–pCRP complex or pCRP–bilayer complex. The complexes were geometry optimized for at least 2,000 iterations (or until the gradient of successive iterations was <0.05 kcal mol^−1^·Å) using the molecular mechanics MMFF94s force field and partial atomic charges, and the conjugate gradient minimization method (all other parameters were at default values) to relieve any steric conflicts that may have arisen during the docking or modelling process. The pentameric pCRP* model was constructed by manually moving the individual subunits of pCRP apart until the interacting side chains were >4 Å apart (Fig. 7e) or the globular head of complement C1q (PDB ID: 1PK6; ref. 47) was able to dock inside the inner annular void, bringing the known interacting residues into contact (Fig. 7g)^47^,^48^,^49^. The C1q-pCRP* complex was then manually docked to the model CHO:POPC lipid bilayer and geometry optimized using the protocol described above. A rotamer library of experimentally determined tryptophan side-chain conformations was used to place Trp205 in the pCRP* model into a solvent-exposed orientation (Supplementary Fig. 9C), consistent with previous tryptophan fluorescence emission studies^13^. One subunit from the pCRP crystal structure was used to generate the unfolded mCRP molecule (Supplementary Fig. 9D). The number of salt bridges, hydrogen bonds and nonpolar interactions at the pCRP intersubunit interface were calculated using PISA v.1.51 (http://www.ebi.ac.uk/msd-srv/prot_int/pistart.html). p*K*_a_ predictions for the acidic residues located at the intersubunit interface were performed using the Depth web server (http://mspc.bii.a-star.edu.sg/depth/)^68^. Figures were created using PyMOL version 1.7.4.1 (Schrodinger LLC; http://www.pymol.org).

### CRP fluorescence analysis

Fluorescence quenching experiments were performed in 20 mM Tris (pH 7.4) containing 100 mM NaCl and 2 mM CaCl_2_ (TBSCa) at a protein concentration of 5 μg ml^−1^. In the samples containing liposomes (40% CHO, 40% PC and 20% LPC), premade liposomes (see below for information on liposome preparation) were included at a final concentration of 100 μg ml^−1^. Samples were prepared by the addition of aliquots of a 2 M KI stock containing 10 mM Na_2_S_2_O_3_ with an equivalent stock of KCl used to correct for any salt-induced effects. Fluorescence intensity was measured on a Perkin-Elmer EnSpire plate reader with an excitation wavelength of 295 nm and an emission wavelength of 340 nm. Fluorescence quenching data were analysed as previously described^69^. Liposomes were prepared from Egg-phosphatidylcholine (eggPC) and LPC (Avanti Polar Lipid). The lipids were dissolved in chloroform/methanol (3/1, v v^−1^) and mixed in a ratio of 4:1 (eggPC:LPC; mol), were dried under nitrogen gas and then evaporated by vacuum overnight. The lipid was then hydrated with MilliQ water to a concentration of 200 μg ml^−1^ at 60 °C, shaking them occasionally for 2 h. The lipid mixture was sonicated for 30 s intervals 10 times and passed through two pieces of polycarbonate membrane (0.2 μm pore size; Avanti Polar Lipids) with a Mini-Extruder (Avanti Polar Lipids). Thirty passes were performed with each membrane. Liposomes were stored at 4 °C until use.

### Microvesicle phagocytosis

Engulfment of microvesicles by human macrophages was analysed by a modified flow cytometric assay that has been described previously^50^. THP-1 cells were labelled with CMFDA according to the manufacturer's instructions before microvesicle purification. Labelled microvesicles (25 μg ml^−1^) were incubated with 0.3 × 10^6^ adherent macrophages for 2 h at 37 °C in the presence of 10% autologous human serum and 25 μg ml^−1^ of either pCRP or mCRP. After washing, cells were released, stained with anti-CD14-PE antibodies (1:100) and analysed by two-colour flow cytometry. Macrophages were gated by forward, side scatter and CD14 positivity. Unlabelled microvesicles served as a negative control. The percentage of macrophages that bound or ingested labelled microvesicles was calculated.

### CD spectroscopy

Generating mCRP by treating pCRP with 8 M urea in the presence of 10 mM EDTA for 2 h at 37 °C or by heating pCRP for 5 min at 95 °C in 0.1% SDS results in an unfolded protein similar in size, solubility, antigenicity and *in vitro* activity as the recombinant human mCRP C36A,C97A-double mutant protein^31^. The CD spectra of pCRP and recombinant C36A,C97A mCRP (both at 190 μg ml^−1^) were recorded at 25 °C using a Jasco J-815 CD spectrometer equipped with a Peltier-type temperature control system (JASCO model PTC-423S/15) and interfaced to a personal computer. The CD spectra were measured from 190 nm to 260 nm every 0.1 nm with 5 s averaging per point and a 2 nm bandwidth. A 0.1 cm path length cell was used for obtaining the spectra. The CD spectra were signal averaged by adding four scans and baseline corrected. The difference spectrum was obtained by subtracting the averaged pCRP curve from the averaged mCRP curve with the Jasco suite of software.

### Statistical analysis

Statistical analysis was performed with GraphPad Prism v.5.0 (GraphPad Software, La Jolla, CA, USA). For comparison of two groups, a two-tailed *t*-test was employed. A *P* value of <0.05 was considered statistically significant. All experiments were performed at least three times. The data are expressed as mean±standard error of the mean (s.e.m.) or standard deviation (s.d.) when indicated. A one-way analysis of variance to compare effects of different treatments was used, if more than two groups were compared. In case of significance, Tukey's test was used for pairwise comparison. Only significant results for both analysis of variance and Turkey's test are presented.

### Data availability

All relevant data are available from the authors on request.

## Additional information

**How to cite this article:** Braig, D. *et al*. Transitional changes in the CRP structure lead to the exposure of proinflammatory binding sites. *Nat. Commun.* **8,** 14188 doi: 10.1038/ncomms14188 (2017).

**Publisher's note:** Springer Nature remains neutral with regard to jurisdictional claims in published maps and institutional affiliations.

## Supplementary Material

Supplementary Information Supplementary Figures 1-14, Supplementary Table 1 and Supplementary References.
