# Supplementary material for: Transitional changes in the CRP structure lead to the exposure of proinflammatory binding sites
Source: Nat Commun. 2017 Jan 23;8:14188. doi: 10.1038/ncomms14188 (PMC5264208; doi:10.1038/ncomms14188)
Supplement: Supplementary Information — Supplementary Figures 1-14, Supplementary Table 1 and Supplementary References. [file ncomms14188-s1.pdf]

Supplementary Figure 1

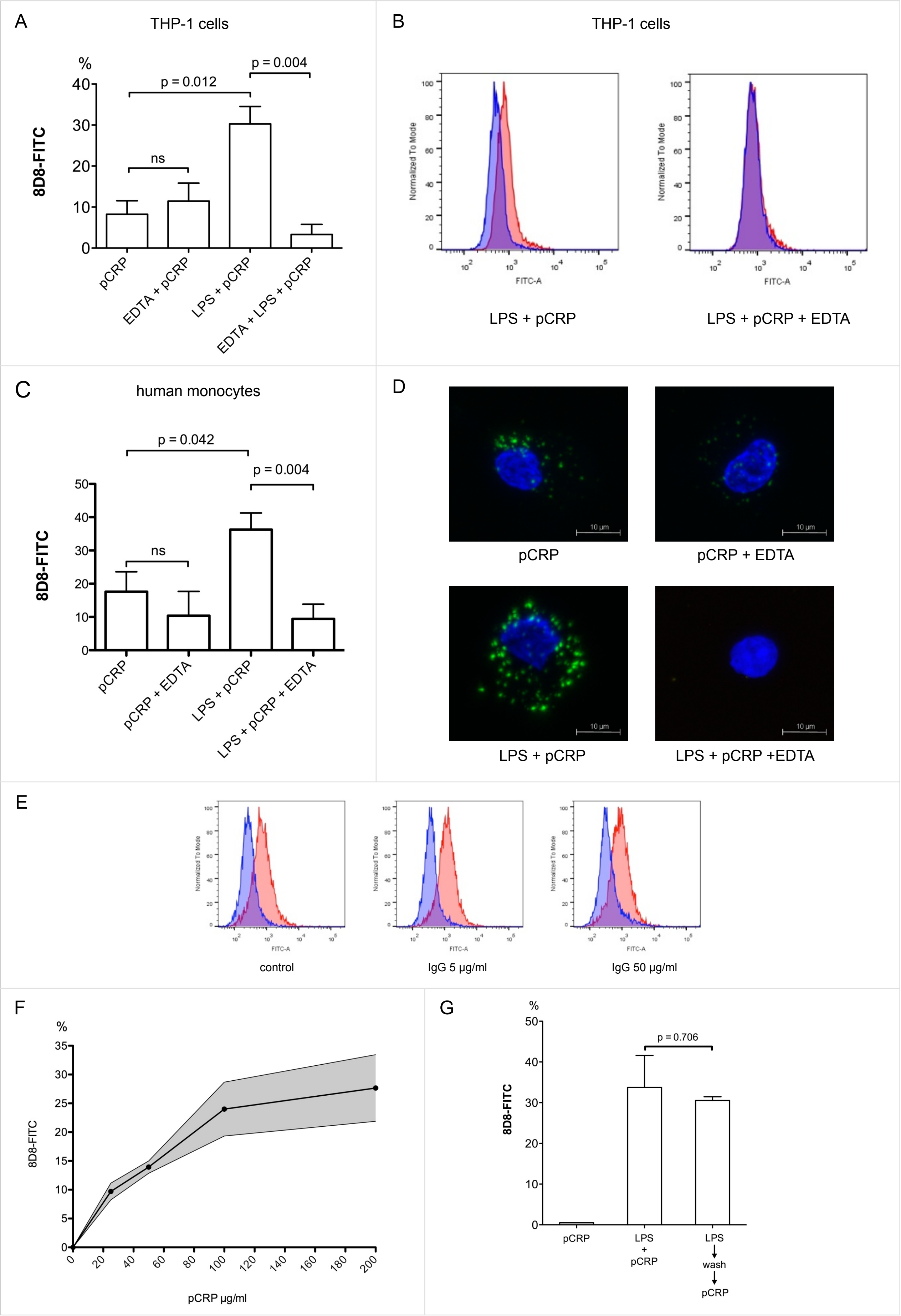

Supplementary Figure 1: Binding of pCRP to THP-1 cells and human monocytes. (A) Binding of pCRP to THP-1 cells was assessed by flow cytometry with conformation specific antibodies for pCRP (anti-pCRP-8D8) and a FITC labeled secondary antibody. EDTA significantly reduced binding of pCRP to LPS-activated THP-1 cells, indicating a specific, Ca^{2+}-dependent interaction. Displayed are mean values and the SEM (n=3). p-values were calculated with a paired t-test. **(B)** Representative fluorograms of LPS-activated THP-1 cells in the absence (blue) and presence (red) of pCRP. Ca^{2+} was depleted by EDTA in the experiment depicted in the right graph. **(C)** Binding of pCRP to human monocytes was assessed by flow cytometry with conformation specific antibodies for pCRP (anti-pCRP-8D8) and a FITC labeled secondary antibody. EDTA significantly reduced binding of pCRP to activated monocytes, indicating a specific, Ca^{2+}-dependent interaction. Displayed are mean values and the SEM (n=5). p-values were calculated with a paired t-test. **(D)** Representative confocal fluorescence microscopy pictures (3D reconstruction) of human monocytes. Cells were treated as in (C). The nuclei were stained with DAPI (blue), pCRP with anti-pCRP-8D8 and a FITC labeled secondary antibody (green). Scale bars depict 10 µm. **(E)** Binding of pCRP to LPS-activated human monocytes was analysed by flow cytometry. Cells were treated as in (C), however, indicated amounts of purified human IgG were added, which binds to human Fcγ receptors. Neither 5 µg/ml nor 50 µg/ml of purified human IgG had an influence on the LPS induced pCRP-cell interaction. **(F)** LPS-activated human monocytes were incubated with increasing concentrations of pCRP and analysed by flow cytometry as described under (C). Binding was mono-exponential, saturable and concentration-dependent within the physiological range of pCRP concentrations during inflammatory reactions. Displayed are the mean values and the SEM (n=3). **(G)** Binding of pCRP to LPS stimulated THP-1 cells was determined by flow cytometry. Cells were either treated as described under (A) or washed in PBS after LPS stimulation before they were incubated with pCRP. Washing did not significantly alter binding of pCRP to activated THP-1 cells. Displayed are the mean values and the SEM (n=3). p-values were calculated with a paired t-test.

Supplementary Figure 2

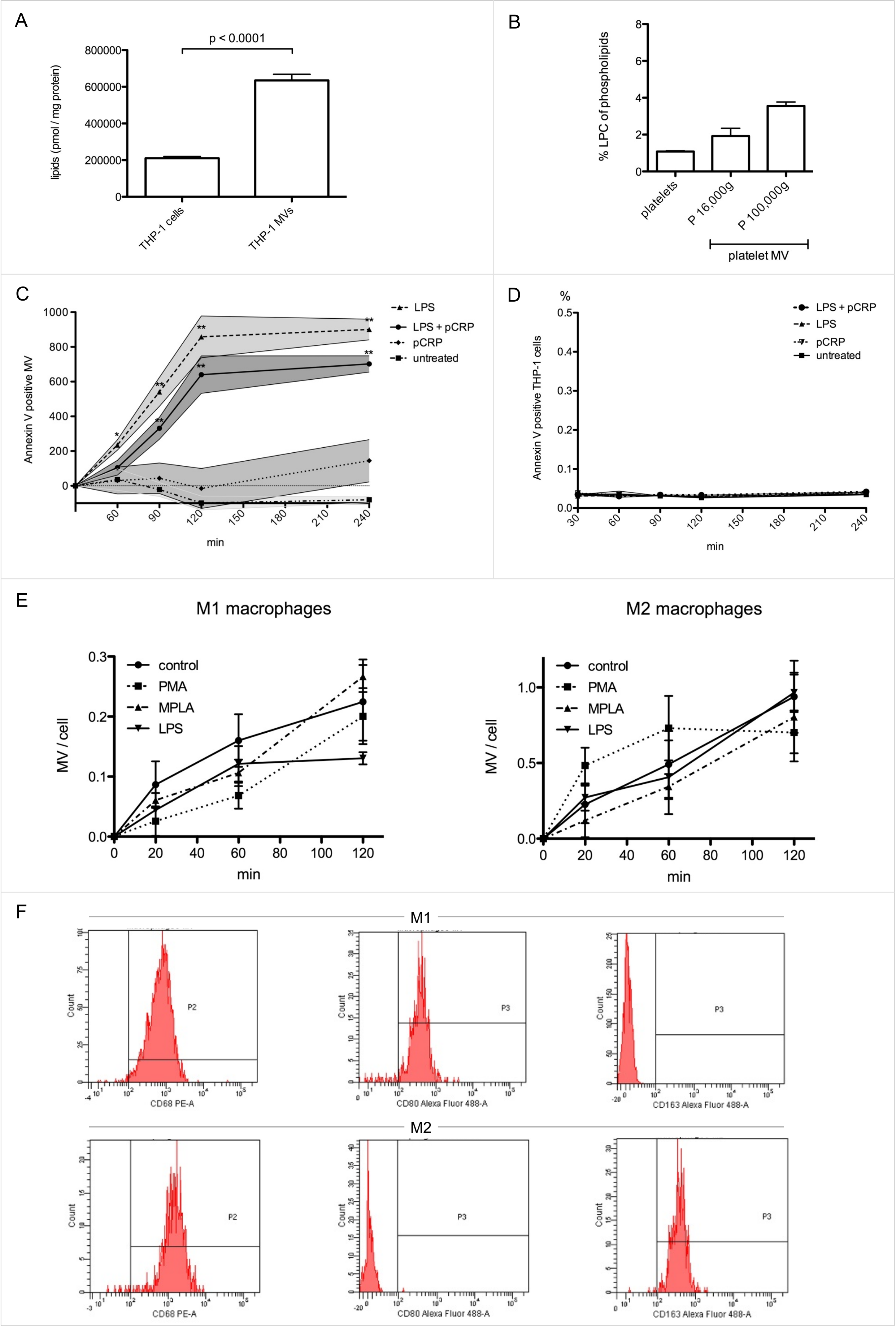

Supplementary Figure 2: Release of microvesicles by activated cells. (A) Depicts the lipid to protein ratios of THP-1 cells and THP-1 microvesicles (16,000 g). THP-1 microvesicles have a significantly higher lipid/protein ratio when compared to their cells of origin. Indicated are means and the SEM (n=9). p-values were calculated with an unpaired t-test. **(B)** LPC content of platelet derived microvesicles. Platelets were stimulated with ADP and the cell-free supernatant subjected to differential centrifugation. Smaller microvesicles contain increased amounts of LPC, which induces a positive membrane curvature. Displayed are means and SEM (n=2). **(C)** THP-1 cells were incubated with LPS and pCRP where indicated. Released microvesicles were counted by flow cytometry at indicated time points. Background levels at t=0 were subtracted (n=3). * indicates a p-value of <0.05 and ** a p-value of <0.01 compared to unstimulated cells. p-values were calculated with one-way ANOVA. **(D)** Binding of the apoptotic marker Annexin V to THP-1 cells was further determined in the course of the experiment. Neither LPS nor pCRP led to increased apoptosis. Displayed are means and SEM (n=3). **(E)** Microvesicle release by M1- and M2-macrophages in the presence of PMA, MPLA and LPS. Stimulation did not lead to enhanced microvesicle production. Displayed are means and SEM (n=3). **(F)** Representative fluorograms of M1- and M2- macrophage differentiation markers. Both populations stain positive for CD 68. M1 cells additionally stain positive for CD 80 and M2 macrophages for CD163.

Supplementary Figure 3

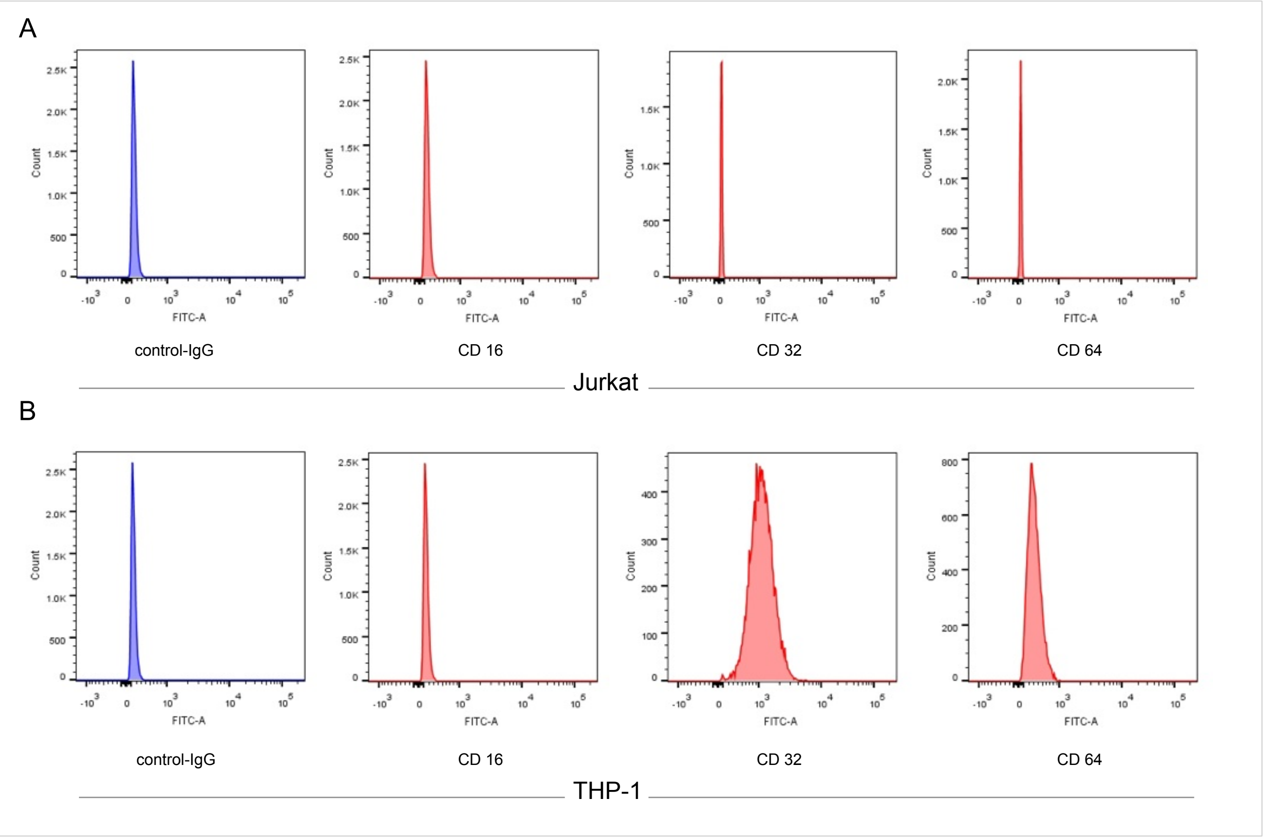

Supplementary Figure 3: Expression of different Fcγ receptors (CD16, CD32, CD64) implicated in CRP binding. Receptor expression was analysed by flow cytometry on Jurkat **(A)** and THP-1 **(B)** cells. The isotype control is displayed in blue and the target antibodies in red. Jurkat cells do not express CD16, CD32 and CD64. In contrast, CD32 and CD64 could be detected on THP-1 cells.

Supplementary Figure 4

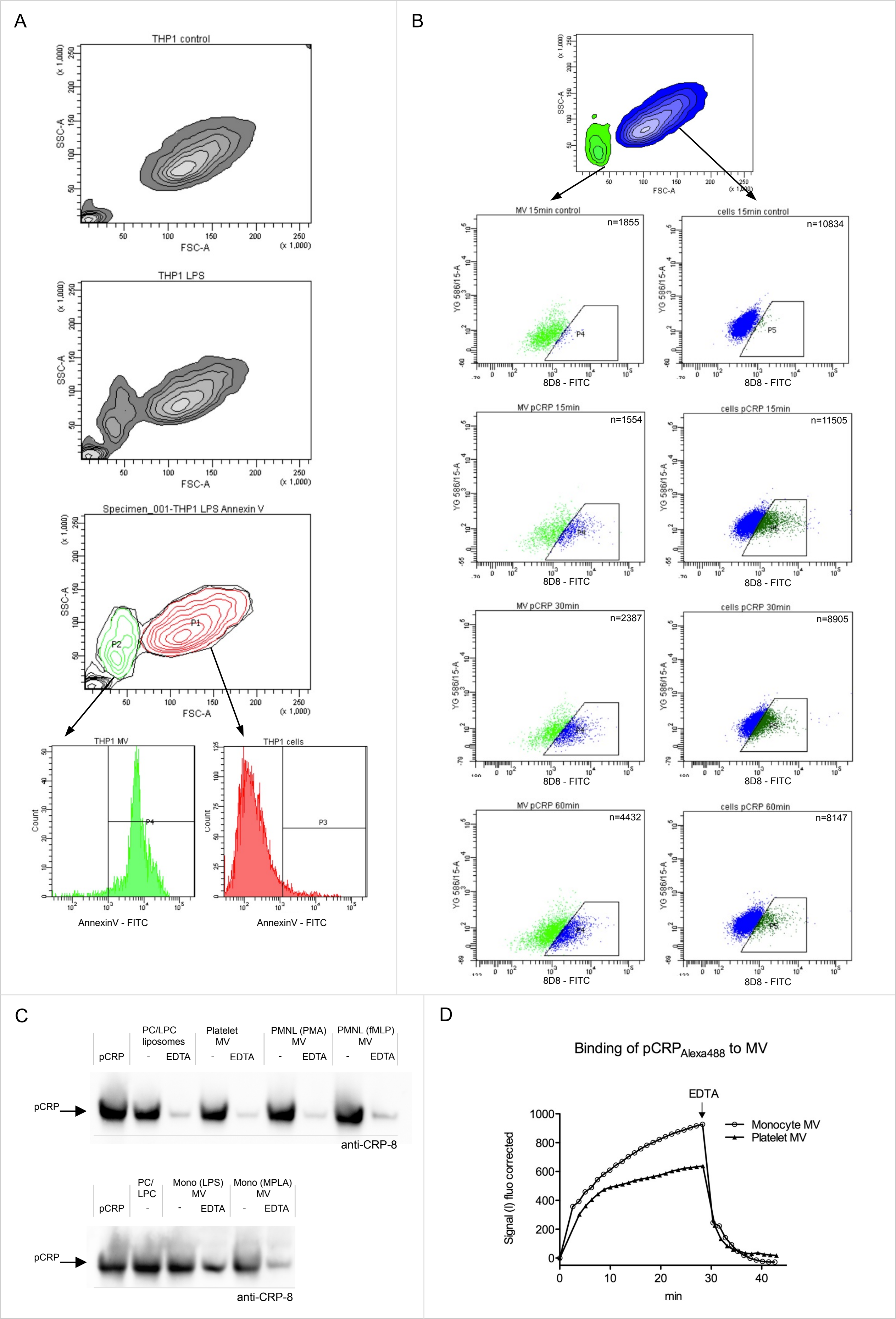

Supplementary Figure 4: (A + B) Gating strategy to determine CRP binding to THP-1 cells and THP-1 microvesicles by flow cytometry. **(A)** THP-1 cells release microvesicles, which can be detected as a separate population in FSC-A/SSC-A upon LPS stimulation (green). In contrast to intact cells (red), microvesicles expose phosphatidylserine on their outer membrane surface, which binds Annexin V - FITC. **(B)** Microvesicle- and cell-bound CRP can be detected with anti-pCRP-8D8 and anti-mCRP-9C9 antibodies by gating on the respective population. Whereas THP-1 cells show only transient binding of pCRP (with subsequent release on pCRP-covered microvesicles), the amount of microvesicle-associated pCRP increases over time. Notice the increasing count of microvesicles over time after LPS stimulation. “n” depicts the whole number of cells or microvesicles in the respective figure. **(C)** Binding of pCRP to different microvesicle species was analysed by BN-PAGE. Microvesicles were purified from stimulated monocytes (Mono), polymorphonuclear leukocytes (PMNL) and platelets. Purified microvesicles were incubated with pCRP or pCRP-EDTA. Subsequently microvesicle-bound CRP was pelleted by ultracentrifugation and separated on BN-PAGE. CRP was detected by anti-CRP-8 antibodies after in-gel denaturation and Western blot. **(D)** Kinetics of the pCRP-microvesicle interaction. The association of Alexa488 labeled pCRP with immobilized microvesicles of monocytic and platelet origin was determined by repeated fluorescence measurements. Dissociation was observed after adding EDTA.

Supplementary Figure 5

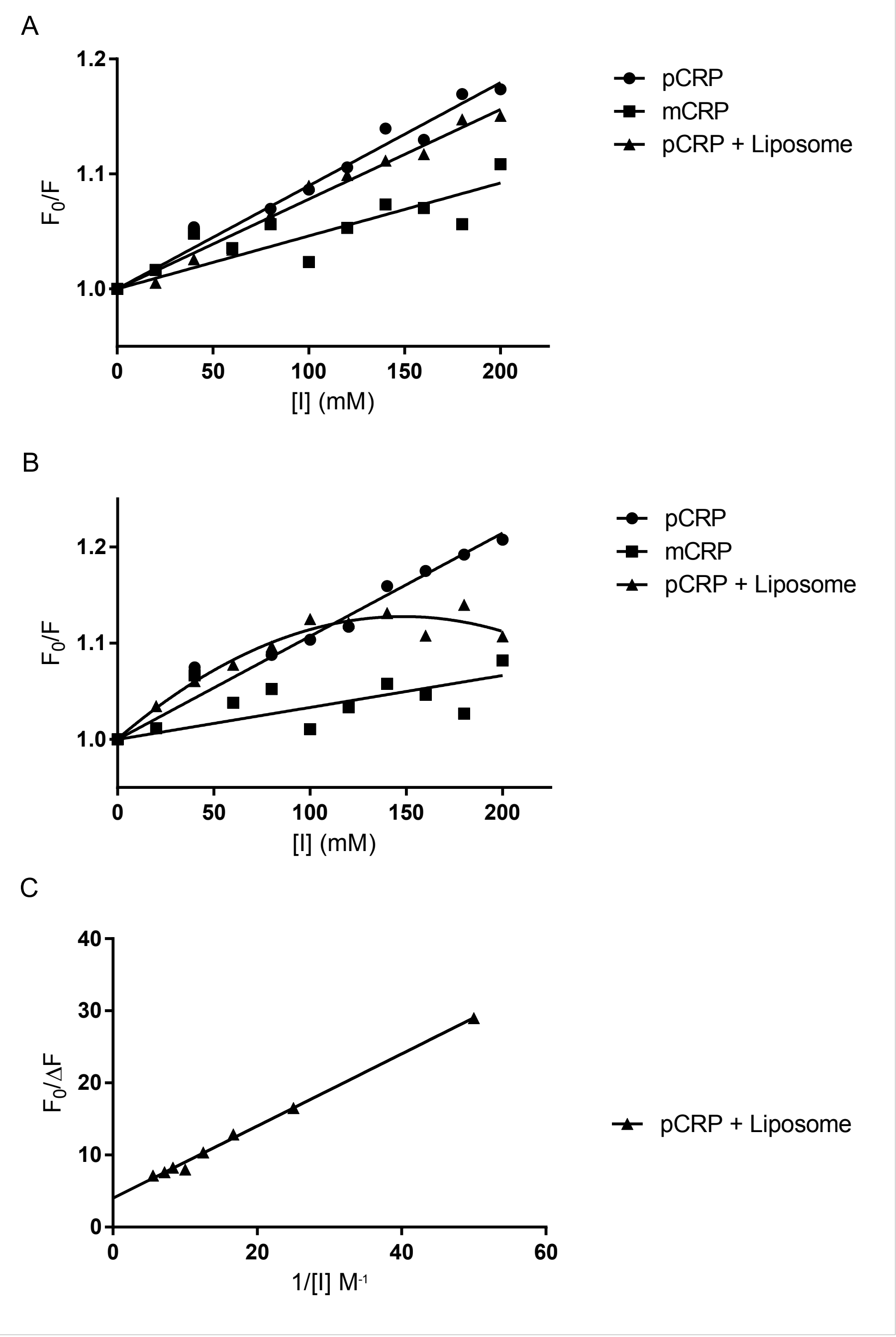

Supplementary Figure 5: CRP fluorescence analysis. (A) Stern-Volmer plot for fluorescence quenching by iodide of freshly prepared CRP samples. **(B)** Stern-Volmer plot for fluorescence quenching of CRP samples after overnight incubation. Note that the curve drawn to the pCRP + liposome sample is an arbitrary polynomial. **(C)** Modified Stern-Volmer plot for fluorescence quenching of pCRP + lipid after overnight incubation. There is a clear difference in the accessibility of the tryptophan residues to the charged quencher iodide when comparing pCRP and mCRP **(A)**. Shown is a representative graph from n=3 independent experiments.

The lower KSV for mCRP suggests that the quenching agent is more easily able to interact with and hence quench the fluorescence of the tryptophan residues as compared to pCRP. Freshly prepared pCRP and pCRP + liposome samples, however, appear to be effectively identical in their accessibility to quenching by iodide. Published data suggests that the formation of a pCRP-liposome complex takes some time, with fluorescence data indicating a stable state is not formed for around 2 hours[1]. Allowing this process to proceed by incubating the samples overnight shows that pCRP remains unchanged, but the pCRP-liposome complex that forms has altered the accessibility of the tryptophan residues in the protein as indicated by a non-linear Stern-Volmer plot **(B)**. A modified Stern-Volmer plot **(C)** indicates that ~25% of the tryptophan fluorescence is easily accessible to iodide with the remaining residues protected.

Supplementary Figure 6

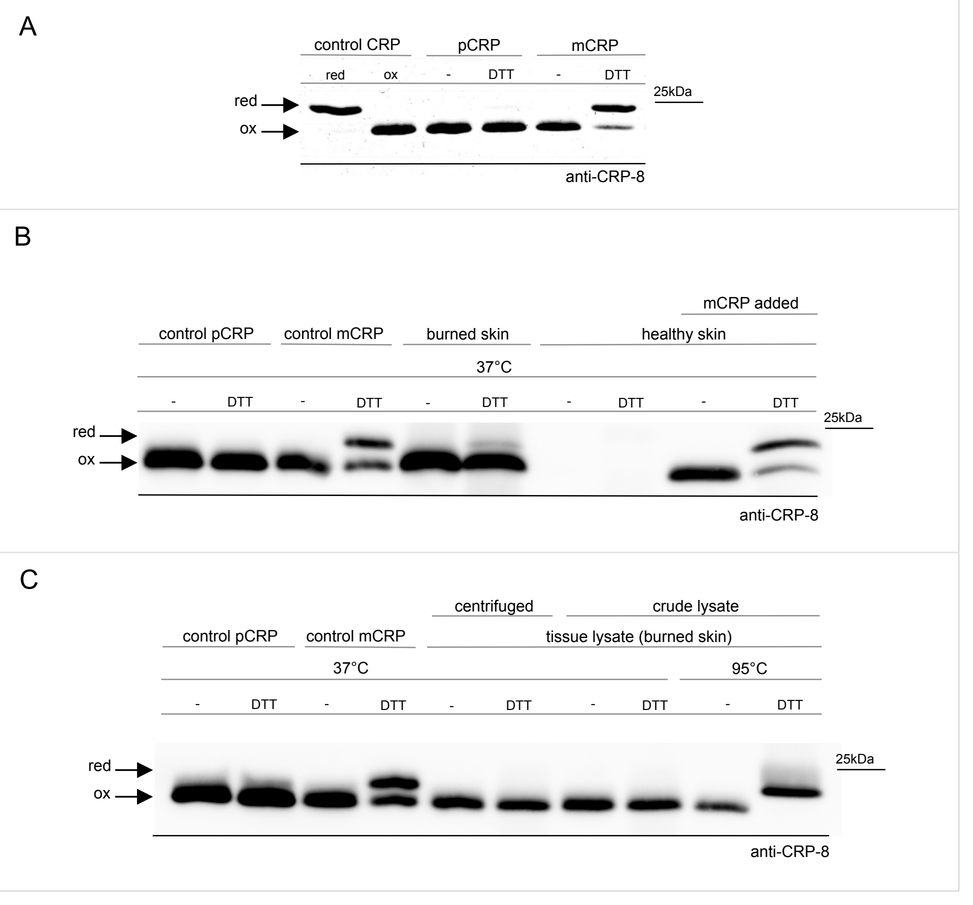

Supplementary Figure 6: Accessibility of the intra-subunit disulfide bond in mCRP. (A) Accessibility of the C36-C97 disulfide bond (location shown in Suppl. Fig. 9C) in pCRP and mCRP. CRP isoforms were incubated with DTT. The reaction was stopped with NEM and the proteins separated on oxidizing SDS-PAGE. CRP was detected with anti-CRP-8 antibodies after Western blot. Consistent with previous reports, we did not observe reduction of the intra-subunit disulfide bond in pCRP, whereas it was efficiently reduced in mCRP. **(B)** To exclude the possibility that components within the tissue lysate inhibited the detection of mCRP, control mCRP was spiked into lysates of healthy skin. We observed efficient reduction under these conditions, indicating that mCRP can reliably be detected in tissue lysates by this assay. Experiments were conducted as described above. **(C)** As mCRP has a tendency to aggregate under physiological buffer conditions we also investigated whether mCRP is lost during clearing of tissue lysates by centrifugation. Tissue was lysed according to our standard protocol and the crude extract was then analyzed without further centrifugation. Again, we observed only minimal reduction of tissue deposited CRP in our assay. Samples were treated as in (B), however, the tissue lysate was not cleared by centrifugation when indicated (*crude lysate*).

Supplementary Figure 7

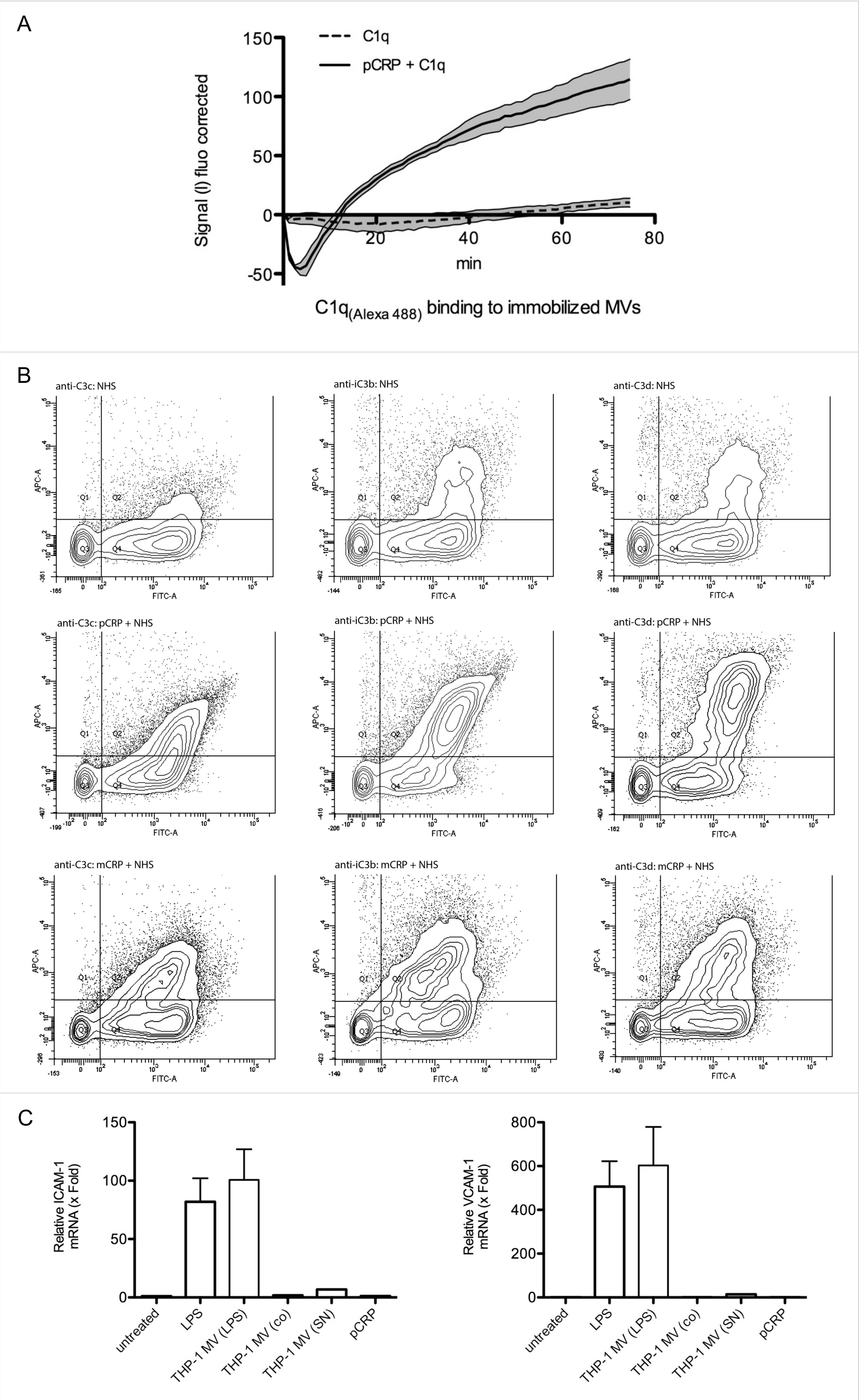

Supplementary Figure 7: (A) Binding kinetics of C1q to immobilized microvesicles. Alexa488 labeled C1q was incubated with immobilized microvesicles in the absence and presence of pCRP. Signal intensity was continuously measured from the microvesicle area and a control area without microvesicles thus recording binding events in real-time. Initially, fast association of Alexa488-C1q to the reference area (5% BSA) was observed in the presence of pCRP, which probably reflects the ability of pCRP to bind to albumin. This was followed by a steady increase in the fluorescence signal, which reflects binding to the microvesicle-covered area. Binding kinetics are similar to the association of CMFDA labeled microvesicles to immobilized C1q (Fig. 6 A). Shaded areas represent SEM (n=3). **(B) Gating strategy to determine C3 deposition on microvesicles by flow cytometry.** CMFDA labeled THP-1 microvesicles (positive in FITC channel) were incubated with normal human sera (NHS) after pre-incubation with pCRP or mCRP. Deposited C3 and its cleavage products were stained with antibodies directed against C3c, iC3b (neoepitope specific), C3d and a second APC labeled antibody. The percentage of C3 positive microvesicles was determined by calculating Q2/(Q2 + Q4). Q3 depicts unlabeled microvesicles and background due to “instrument noise”. pCRP led to increased deposition of complement C3 components. **(C) Expression of VCAM-1 and ICAM-1 mRNA in HUVECs.** Expression was determined by qRT-PCR after treatment with LPS, different microvesicle preparations and pCRP. Microvesicles were purified from the supernatant of activated THP-1 cells (*THP-1 MV (LPS)*) and untreated THP-1 cells (*THP-1 MV (co))*. In addition, the supernatant of the last wash step after microvesicle preparation from activated THP-1 cells (*THP-1 MV (SN))* and pCRP was used. Displayed are mean values and the SEM (n=3).

Supplementary Figure 8

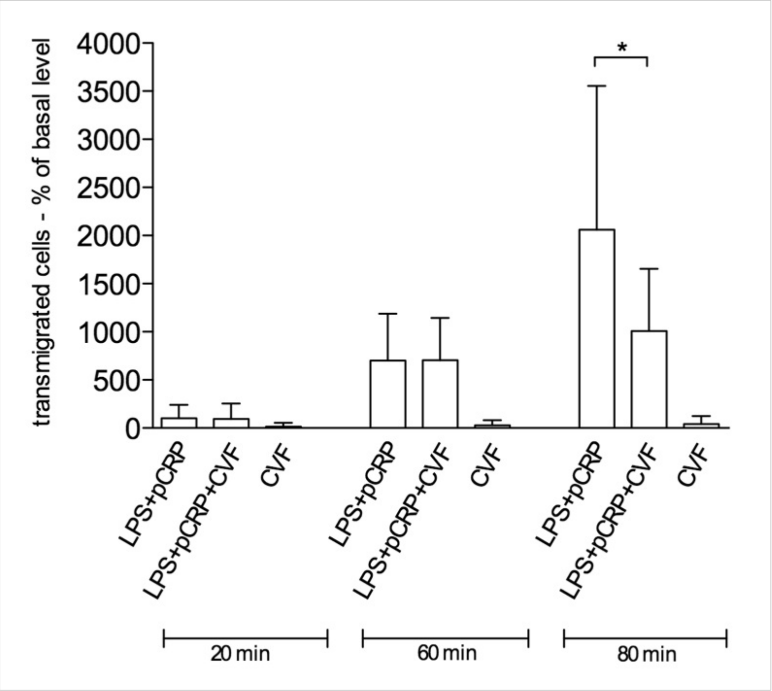

Supplementary Figure 8: Leukocyte transmigration in complement-depleted rats. Leukocyte transmigration was quantified by intravital microscopy of rat cremaster muscle under superfusion with LPS (25 ng/ml) and i.v. application of pCRP (25 µg/ml) in complement-depleted rats. The complement system was depleted by i.p. injection of cobra venom factor (CVF; 250 U/kg body weight) 24 hours prior to intravital imaging. CVF significantly reduces the CRP-mediated leukocyte transmigration at 80 min. Displayed are means and SEM. Statistical analysis was performed with one-way ANOVA, * $p<0.05$, ($n\geq4$ in each group).

Supplementary Figure 9

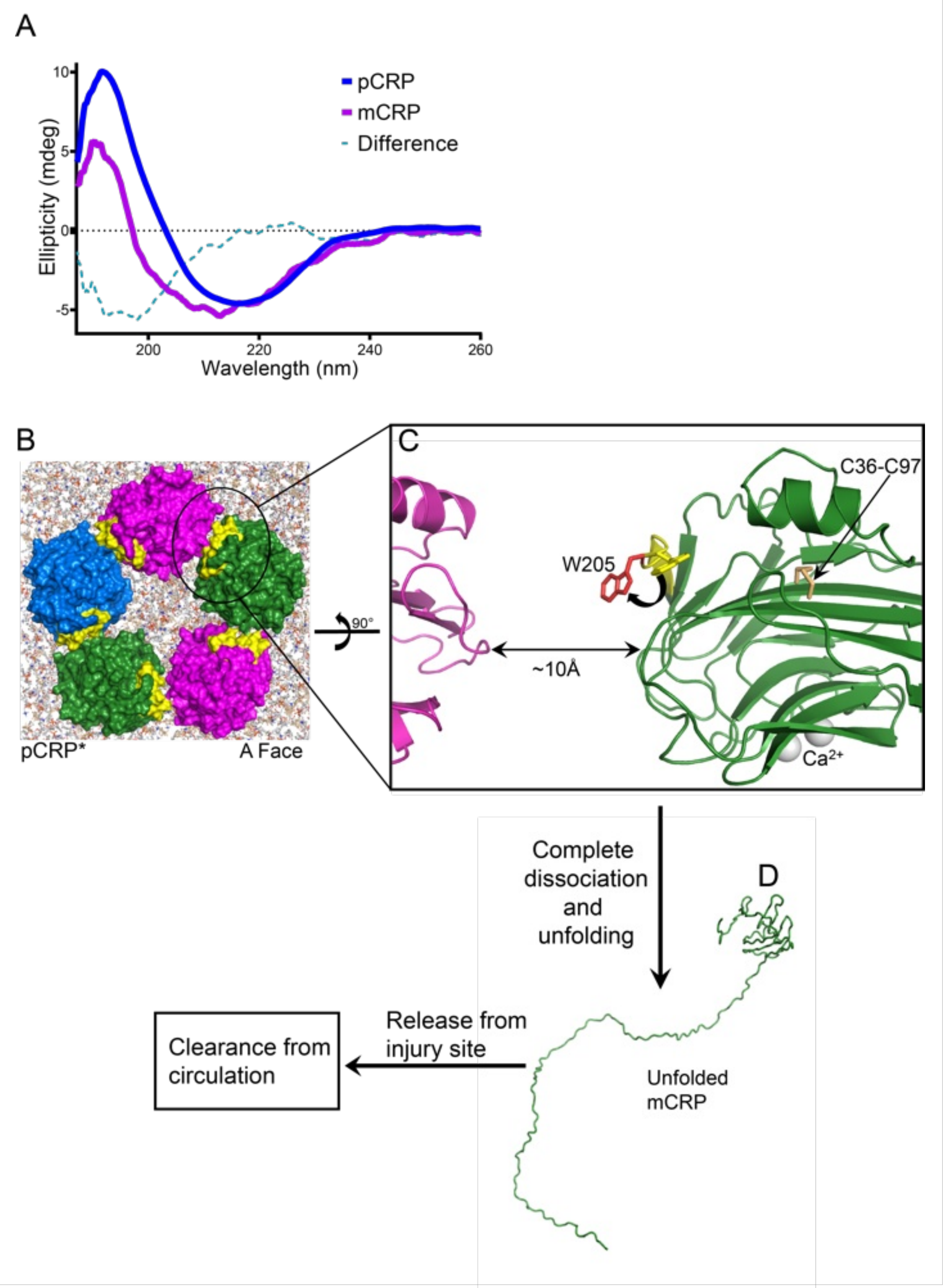

Supplementary Figure 9: Comparison of the UV CD spectra of pCRP and mCRP and model illustrating pCRP* dissociation to mCRP. **(A)** UV CD spectra of pCRP (solid blue line) and mCRP (solid purple line) in 20 mM sodium phosphate buffer (pH 7.5) and 25 mM NaCl. The difference between the two curves (Δ = mCRP – pCRP; dashed light blue line) indicates the secondary structure difference between these two CRP isoforms. **(B)** Model of pentameric pCRP* on a cholesterol:POPC lipid bilayer, same view and coloring as Fig. 7E. The antigenic neo-epitope for the mCRP-specific antibody anti-mCRP-9C9, i.e. CRP residues 199-206 (yellow), is exposed at the inter-subunit interface. **(C)** Close up view of the inter-subunit interface after a rotation of +90° about the X-axis. The lipid bilayer is not shown. pCRP* is shown in cartoon format and the distance between the protein backbone of adjacent CRP subunits is indicated. At this distance the electrostatic interactions (salt bridges and hydrogen bonds) between amino acid side-chains on adjacent CRP subunits are extremely weak (distance > 4 Å). Three main options are available to pCRP*: (i) maintain the current structure (eg. for C1q binding, Fig. 7G and H); (ii) reform the native pCRP structure (Fig. 7C and D) and (iii) completely dissociate. The location of the intra-subunit disulfide bond (C36-C97, cream colored sticks) and the two Ca^{2+} ions (grey spheres) are indicated. Trp205 (W205) in the neo-epitope (yellow) is now able to flip out becoming solvent exposed (red W205) and the neo-epitope is accessible for interaction with antibodies anti-mCRP 9C9 or anti-mCRP 3H12. **(D)** Reducing or Ca^{2+}-deficient conditions will result in the breaking of the intra-subunit disulfide bond or removal of the Ca^{2+} ions, respectively, either of which will result in unfolding of the CRP monomer. The unfolded mCRP is then released from the site of injury, or inflammation, for clearance.

Supplementary Figure 10

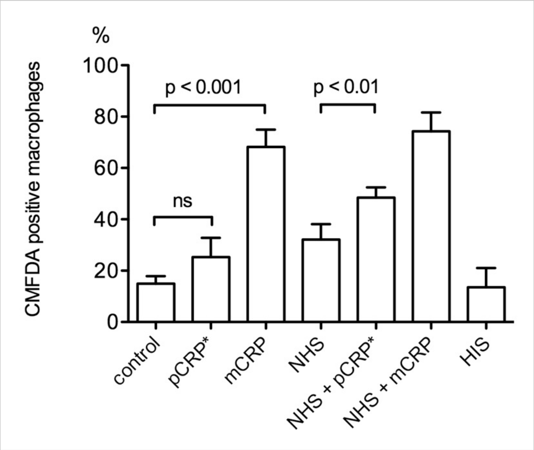

Supplementary Figure 10: Phagocytosis of CMFDA labeled microvesicles by human macrophages was determined by flow cytometry. pCRP* significantly increased uptake of labeled microvesicles in the presence of normal human sera (NHS). mCRP was even more potent, and its effect could not be enhanced by NHS. Displayed are the means and SEM (n≥3 in each group). p-values were calculated with a paired t-test.

Supplementary Figure 11

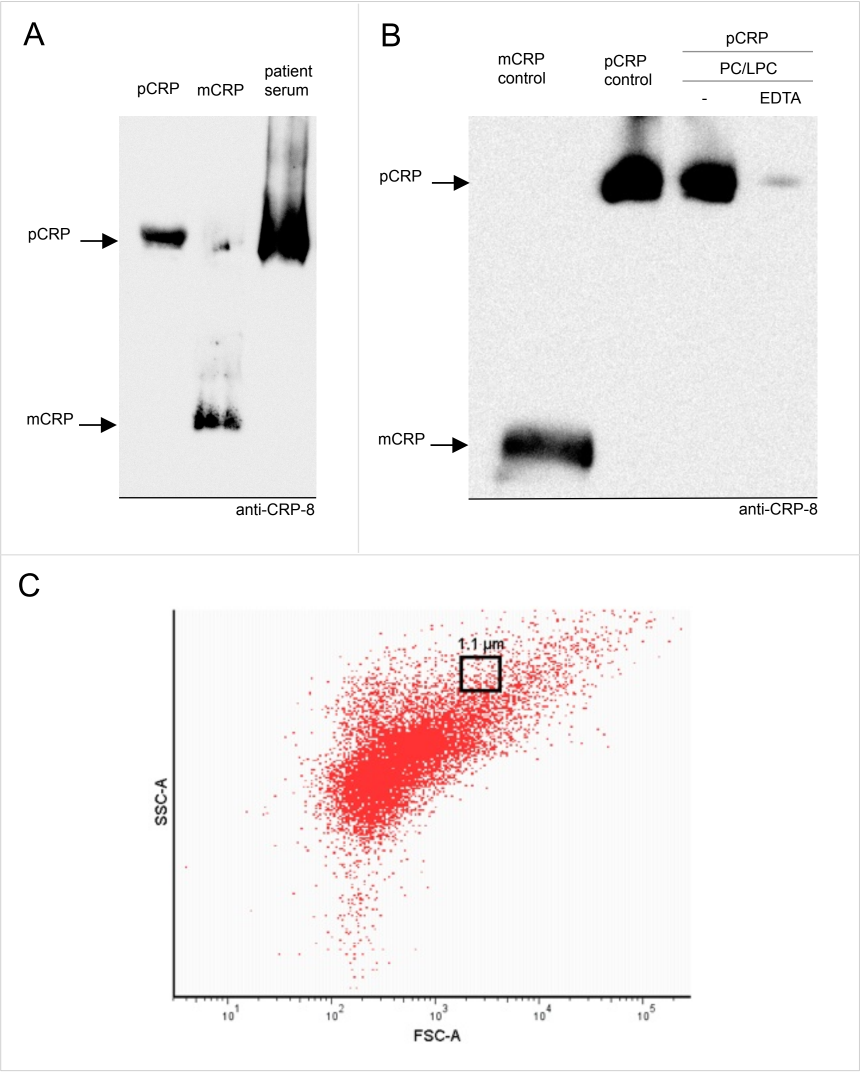

Supplementary Figure 11: CRP isoforms present in burn patient serum, binding of pCRP to liposomes and microvesicle size analysis. (A) Commercial pCRP, urea dissociated pCRP (i.e. mCRP) and serum from a burn patient was separated on BN-PAGE. The gel was briefly boiled in SDS and DTT containing gel buffer, which induces dissociation of pCRP and thus enables uniform detection of CRP isoforms by anti-CRP-8 antibodies after Western blot. Commercial pCRP migrated at the same height as circulating human CRP in serum. No mCRP contamination was observed in commercial pCRP preparations. **(B)** The structural integrity of commercial pCRP was determined by assessing its ability to bind to PC/LPC liposomes. The interaction of pCRP with liposomes was Ca^{2+}-dependent, suggesting a specific interaction between pCRP and exposed phosphocholine head groups of the PC/LPC liposomes. **(C)** Purified microvesicles (P 16,000 g) were analyzed by flow cytometry. Microvesicles were highly pure and the majority of particles had a size < 1.1 µm.

Supplementary Figure 12

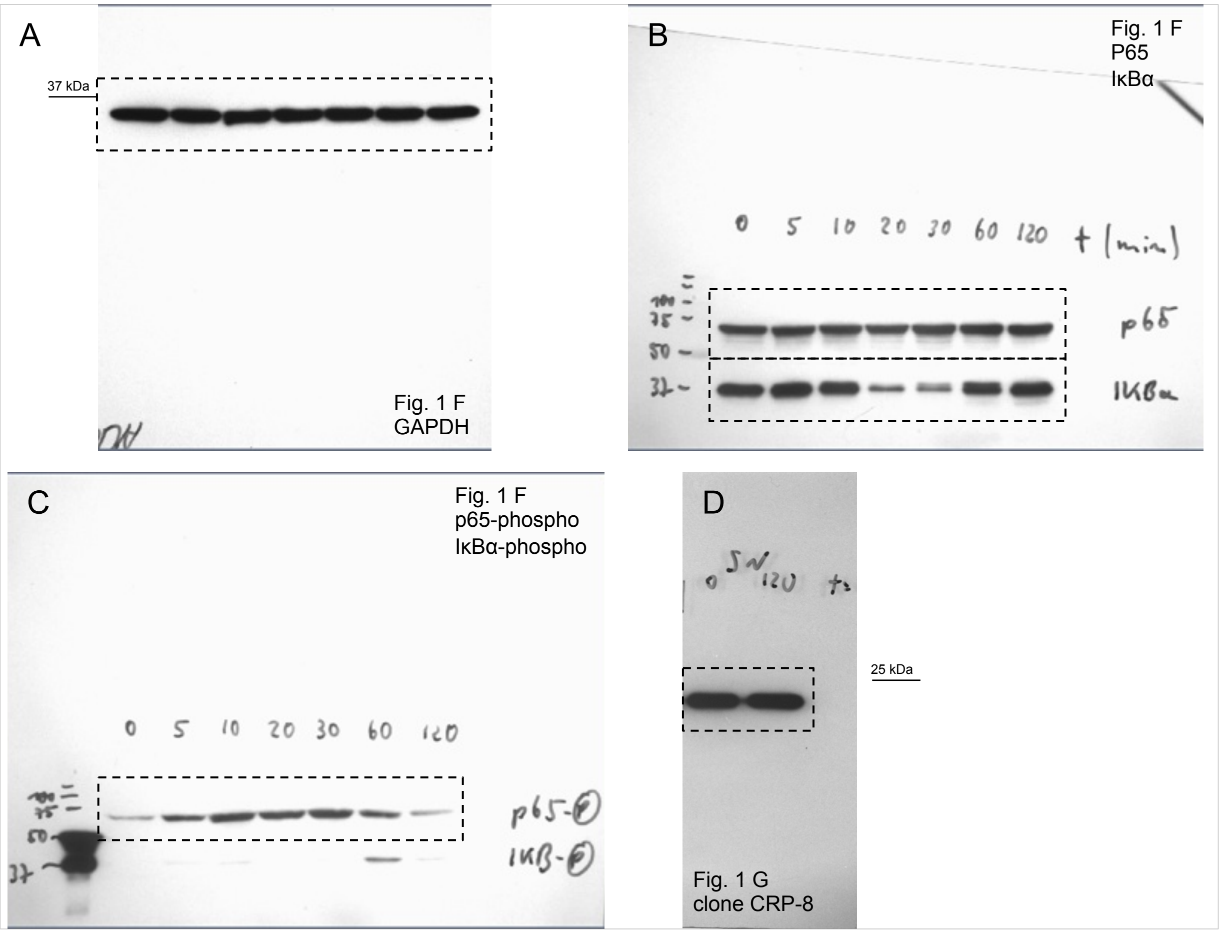

Supplementary Figure 12: Uncropped images for western blots and SDS-PAGE gels. Broken box marks the borders of the final cropped image.

Supplementary Figure 13

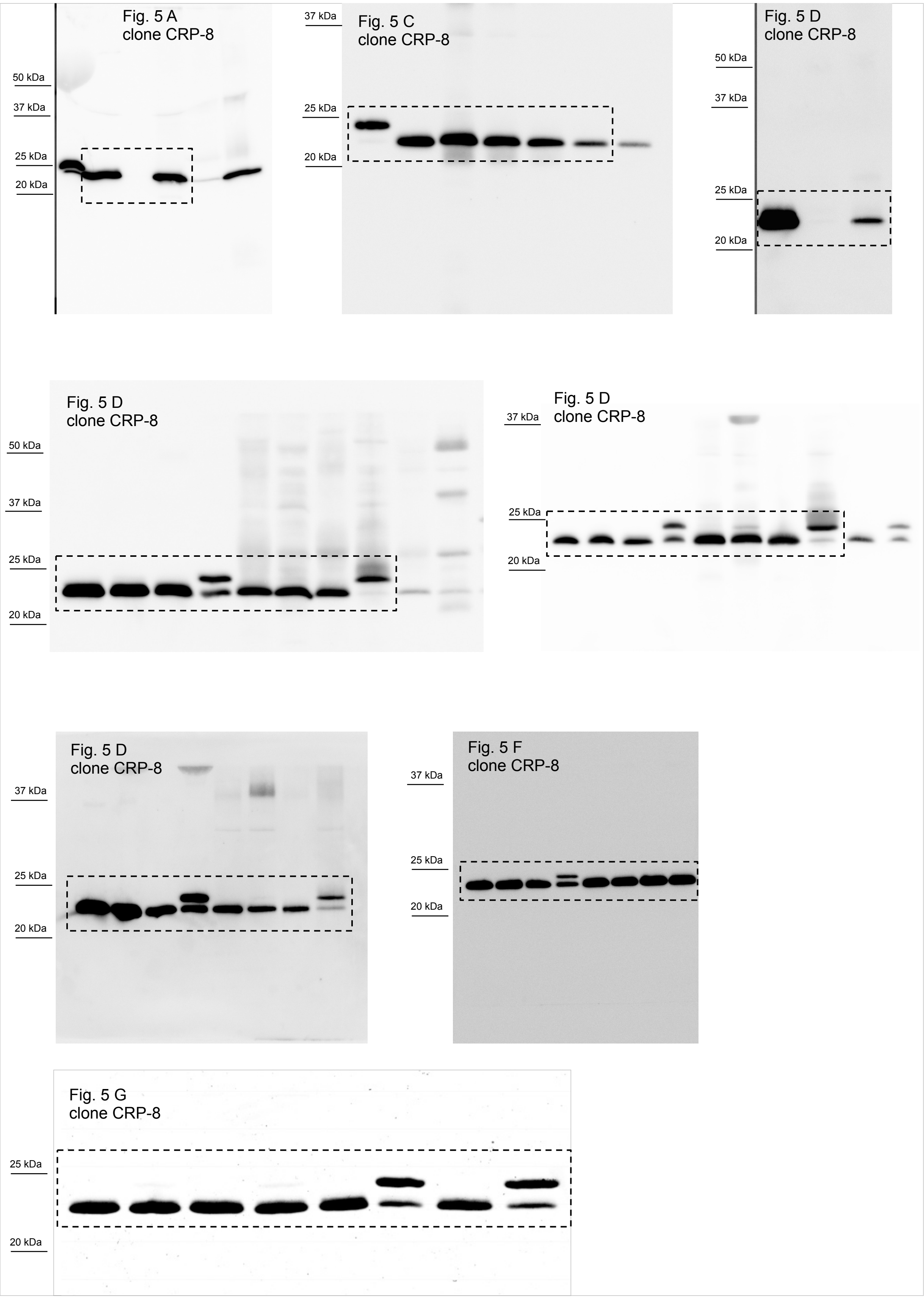

Supplementary Figure 13: Uncropped images for western blots and SDS-PAGE gels. Broken box marks the borders of the final cropped image.

Supplementary Figure 14

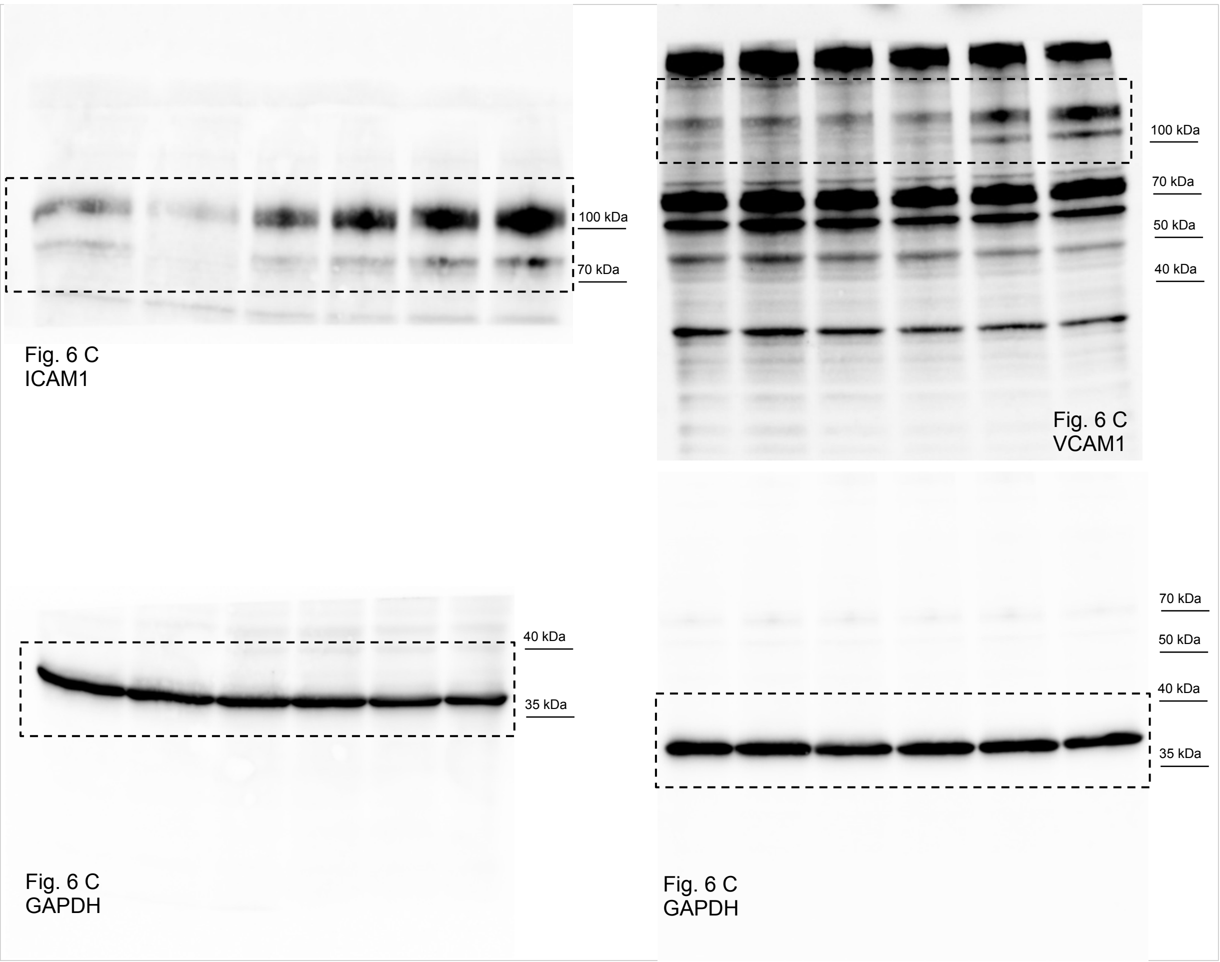

Supplementary Figure 14: Uncropped images for western blots and SDS-PAGE gels. Broken box marks the borders of the final cropped image.

Supplementary Table 1

Patient	Gender	Cause of thermal injury	TBSA (%)	Burn depth	Sample collection	Serum CRP concentration
1	Male	Flame burn	22	Deep dermal	Day 6	312 mg/dl
2	Female	Flame burn	6	Deep dermal to full thickness	Day 4	110 mg/dl
3	Female	Hot water immersion	11	Deep dermal	Day 8	43 mg/dl

Legend to Supplementary Table 1: Patient characteristics. Tissue samples of burn wounds were taken from patients who needed surgical excision of thermally injured skin 4-8 days post trauma. Specimens were snap-frozen in liquid nitrogen and stored at -80°C until analysis. TBSA: total body surface area.

Supplementary References

1. Ji SR*, et al.* Cell membranes and liposomes dissociate C-reactive protein (CRP) to form a new, biologically active structural intermediate: mCRP(m). *FASEB journal : official publication of the Federation of American Societies for Experimental Biology* **21**, 284-294 (2007).
